# Targeted destruction of cancer stem cells using multifunctional magnetic nanoparticles that enable combined hyperthermia and chemotherapy

**DOI:** 10.7150/thno.38989

**Published:** 2020-01-01

**Authors:** Dandan Liu, Yingcai Hong, Yaping Li, Chong Hu, Tak-Chun Yip, Wai-Kin Yu, Yu Zhu, Chi-Chun Fong, Weimao Wang, Siu-Kie Au, Shubin Wang, Mengsu Yang

**Affiliations:** 1 Department of Biomedical Sciences, City University of Hong Kong, Hong Kong, China.; 2 Key Laboratory of Biochip Technology, Biotech and Health Centre, Shenzhen Research Institute of City University of Hong Kong, Shenzhen 518057, China.; 3 College of Chemistry and Environmental Science, Key Laboratory of Medicinal Chemistry and Molecular Diagnosis of the Ministry of Education, Hebei University, Baoding 071002, China.; 4 Department of Thoracic Surgery, Shenzhen People's Hospital, the Second Clinical Medical College of Jinan University, Shenzhen 510000, China.; 5 Department of Clinical Oncology, Queen Elizabeth Hospital, Hong Kong, China.; 6 Department of Oncology, Peking University Shenzhen Hospital, Shenzhen 518036, China.

**Keywords:** Lung cancer stem cell, Multifunctional nanoparticle, Alternating magnetic field, Thermotherapy and chemotherapy

## Abstract

Cancer stem cells (CSCs) have been implicated in cancer recurrence and therapy resistance. Therefore, a CSC-targeted therapy that disrupts the maintenance and survival of CSCs may offer an effective approach in killing tumor cells in primary tumors and preventing the metastasis caused by CSCs. Nanoparticles (NPs)-based thermotherapy and/or chemotherapy are promising therapeutic methods for cancer treatment.

**Methods:** A silica-based multifunctional NP system was present, which encapsulated a chemotherapeutic agent and magnetic cores and coated with a specific antibody against the lung CSCs. The efficacy of this novel therapeutic strategy was systematically studied both *in vitro* and* in vivo* by simultaneous activating the combined thermotherapy and chemotherapy *via* CSC-targeted NPs.

**Results:** These NPs were systematically administered and activated for targeted chemotherapy and thermotherapy by using an externally applied alternating magnetic field (AMF). The antibody-modified NPs targeted to lung CSCs with enhanced cellular uptake *in vitro* and extended accumulation in tumor *in vivo*. Up to 98% of lung CSCs was killed *in vitro* with 30-min application of AMF, due to the combined effects of hyperthermia and chemotherapeutic drug treatment. In *in vivo* models, this combined therapy significantly suppressed tumor growth and metastasis in lung CSC xenograft-bearing mice, with minimal side effects and adverse effects.

**Conclusion:** With good biocompatibility and targeting capability, the nanodrug delivery system may offer a promising clinical platform for the combined thermotherapy and chemotherapy. This work demonstrated the feasibility of developing multifunctional nanomedicine targeting CSCs for effective cancer treatment.

## Introduction

Intratumour heterogeneity of cancer cells presents a great challenge for effective cancer treatment. The existence of cancer stem cells (CSCs) within tumors has been established to have the biological features such as self-renewal, tumor initiating and recurrence, and metastasis, which are associated with the aggressive behaviour of cancer cells 1-3. Recently, clinical evidences have shown the importance of the CSC in many cancers, including in lung cancer 4-9. Lung cancer stem cells (LCSCs) have been isolated from adenocarcinoma and small cell lung cancer (SCLC) patients 10. LCSCs are capable of infinite proliferation* in vitro* and tumor formation* in vivo*, and produced cancers with characteristics identical to those of the original specimens 10-12. These LCSCs are resistant to traditional chemotherapies 13-15. Because of their highly tumorigenic and invasive ability, efficiently eliminating the LCSCs may result in eradicating cancer and preventing relapse and metastasis. However, the conventional chemotherapy primarily aims to kill proliferating cancer cells but not CSCs, which are usually dormant and not proliferating. Thus, developing efficient ways to target and kill or induce the differentiation of the CSCs may represent a potential new approach for cancer treatments.

The idea of combined therapy by integrating two or more therapeutic agents in the form of a multifunctional nanoparticle (MNP) platform has been proposed 16-18, as a strategy to achieve synergistic effects. The combination of chemotherapy and thermotherapy is a feasible way to overcoming the drawbacks of low efficacy, drug resistance, and off-target hyperthermia 16, 19. While combing hyperthermia and chemotherapy has been widely investigated for cancer treatment, its clinical application has been limited due to the problems of unintended heating of normal tissues, nonspecific drug delivery, and consequent side effects. Recently, nanomedicine-based thermotherapy and chemotherapy has not been proposed for CSC-targeted treatment 18-22. However, a thorough study of effective and simple methods to integrate multiple functionalities and properties into a single NP platform is required for CSCs-targeted combined therapy. Compared to a single drug regimen, NPs with a targeting agent could provide targeted and controlled drug delivery with high efficacy and low side effects, and possible imaging capability 23-25. With the rapid development of cancer nanomedicine, the MNP strategy has attracted much attention 26-30. We hypothesized that the co-delivery of hyperthermia and anticancer drug in a single NP may further enhance the effects of cancer therapy. Silica with good biocompatibility, easy modification and resistance to decomposition *in vivo* has been applied as nanocarrier, which can be loaded with a chemotherapeutic drug with an iron oxide core (thermo-therapeutic agent) encapsulated, and modified with a specific cancer marker for targeting tumor cells.

**Figure 1:**
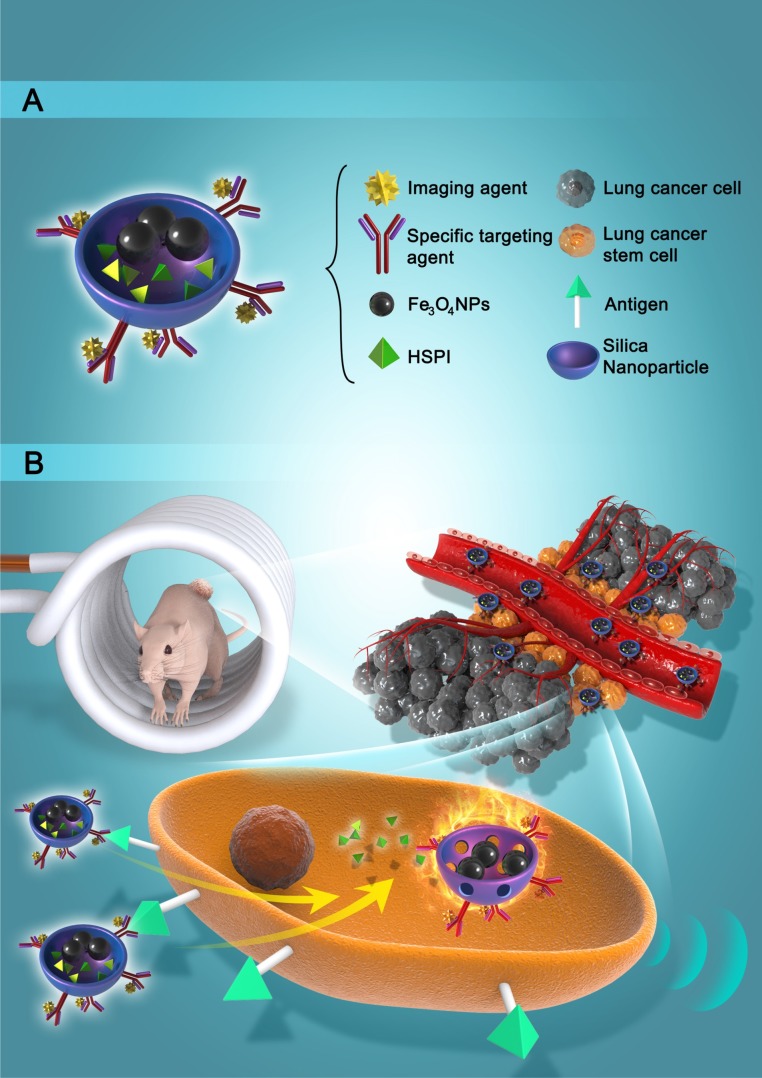
(A) Schematic diagram showed the structure and multifunction of MNPs. (B) LCSCs-targeted combined thermotherapy and chemotherapy by MNPs.

In this work, we designed and developed a highly effective silica-based MNPs platform (CD20-HSPI&Fe_3_O_4_@SiNPs) for combined thermotherapy and chemotherapy targeting cancer stem cells. The MNPs have core/shell structure that the silica shell encapsulating Fe_3_O_4_ nanoparticles as the magnetic core and being loaded with an anticancer drug (heat shock protein inhibitor, HSPI, in this study). The surface of the silica shell was modified with an antibody for a specific marker of LCSCs (CD20). The MNPs were designed target CSCs by applying an alternating magnetic field (AMF) to achieve the combined chemotherapy and thermotherapy. The schematic diagram of the MNPs and its targeted treatment was illustrated in Figure 1. The anti-CD20 labelled with fluorescent dye was conjugated to MNPs to image LCSC targeting performance. The LCSC-targeting ability of the MNPs was evaluated by analysing the cellular uptake and internalization in LCSCs. We further established *in vitro* and *in vivo* LCSC models to test the efficacy of the MNPs in eliminating the LCSCs under an externally applied AMF. The biodistribution and accumulation of MNPs within the tumor region and other organs were studied by *in vivo* and *ex vivo* fluorescence imaging. A mouse lung metastasis model was established to study the effect of MNP-AMF treatment in preventing the metastasis of LCSCs. We believe that the MNPs hold great potential for further development in CSC-targeted cancer treatments due to their optimal antitumor efficacy and high biocompatibility.

## Methods

### Synthesis and Characterization of Multifunctional Nanoparticles

*Preparation of Heat Shock Protein Inhibitor-loaded Silica-coated Fe_3_O_4_ Nanoparticles (HSPI&Fe_3_O_4_@SiNPs)*: The Fe_3_O_4_ NPs and silica-coated Fe_3_O_4_ NPs were synthesized according to the literature procedure 31, 32. Briefly, Fe_3_O_4_ NPs and HSPI were dispersed into a reversed microemulsion solution and stirred for 30 min. Then, tetraethoxy orthosilicate (TEOS) was added to the mixture, followed by the addition of 100 µL aqueous ammonia for the TEOS hydrolysis under stirring for 24 h. After demulsification, the HSPI-loaded Fe_3_O_4_@SiNPs were isolated *via* centrifugation and washed in sequence with ethanol and D.I. water for purification.

*Conjugation of Specific Antibody with HSPI-loaded Fe_3_O_4_@SiNPs*: For specific antibody conjugation, the HSPI-loaded Fe_3_O_4_@SiNPs were modified with carboxyl functional groups, and then PE-labeled anti-CD20 was added into the HSPI-loaded Fe_3_O_4_@SiNPs suspension and stirred for 4 h in the dark.

*Characterization of Multifunctional Nanoparticles*: Emission scanning electron microscope (SEM) and transmission electron microscope (TEM) were used to observe the morphology. The particle size and zeta potentials were determined by a Malvern Zetasizer NanoZS instrument (Malvern, NanoZS). Magnetic properties were measured with a vibrating sample magnetometer (VSM, Model 1600, Digital Measurement System, Newton, MA). The heat generation capabilities of the MNPs were measured using alternating magnetic field (AMF) generator (SPG-06A, Shenzhen Shuangping Power Supply Technologies Co. Ltd.) with 350 kHz frequency and 21 kA/m field strength.

*In vitro Drug Loading and Release*: For HSPI loading, 50 μg/mL HSPI were dispersed into the reaction solution. UV-vis spectrophotometer (PerkinElmer, PE Lamda 750, USA) in the range of 200-800 nm was used to calculate the loading amount of HSPI at 350 nm by the subtraction of free HSPI in the washing solution from the original amount. For drug release, 1 mL medium dispersed-HSPI-loaded Fe_3_O_4_@SiNPs was placed in a dialysis bag (weight cut-off of 10 kDa), and then immersed in 9 mL PBS and kept in an AMF at a constant temperature. The amount of released HSPI was analyzed *via* UV-Visible spectrophotometry (U-3900, Hitachi) and the concentration-absorbance standard equation.

### Lung Cancer Stem Cell Culture and Characterization

*Lung Cancer Stem Cell Culture*: Human lung cancer stem cell line (LCSC) originated from human small cell lung cancer tissue was purchased from Celprogen (Cat# 36107-34, Celprogen, USA). LCSCs were maintained in a serum-free medium (DMEM/F12) supplemented with 2% B27, 10 ng/ml bFGF and EGF, 1% N2, and 1% antibiotic solution and cultured in a humidified atmosphere containing 5% CO_2_ at 37 ^o^C according to the the literature 33-35. The fresh medium was replaced ever three days of culture.

*Immunofluorescence Staining for Stemness Analysis of LCSCs*: The stemness of LCSCs was examined by immunofluorescence staining of surface makers and stemness markers, including PE conjugated-CD20, FITC-conjugated CD15, APC-conjugated ABCG, and Oct4. The nuclei were stained with DAPI. Cells were then washed as described above and observed under the Laser-scanning confocal microscope (Leica SPE).

*Tumor Sphere Formation Assay*: LCSCs (3^rd^ and 10^th^ generation) were plated at a density of 10,000 cells/well in 6-well nonadherent plates (Corning Inc.) in DMEM/F12 cell medium, supplemented with human EGF (10 ng/mL, Invitrogen), N2 (1% v/v, Invitrogen), and human bFGF (10 ng/mL, Invitrogen) for 12 days incubation. Spheres were then washed once with PBS, followed by gentle resuspension.

*In Vivo Study of Tumorigenesis:* All experiments were carried out with BALB/c nude mice, 5-6 weeks old. Mice were maintained in Queen Elizabeth Hospital (Hong Kong, China) under conditions approved by the local animal care committee. To assess the tumorigenic potential of lung cancer stem cells (LCSCs, 3^rd^ generation) and differentiated lung cancer stem cells (dLCSCs, 19^th^ generation), 1×10^4^ LCSCs and dLCSCs were suspended in Matrigel (BD Biosciences) at a ratio of 1:1, and 200 μL of cells was subcutaneously injected into the back of nude mice. The tumor volume was measured every five days after injection and calculated from the formula: length × width × depth × π/6.

### *In Vitro* Cytotoxicity of Multifunctional Nanoparticles and Uptake by LCSCs

*In vitro Cytotoxicity of Multifunctional Nanoparticles:* The cytotoxicity of designed NPs was evaluated by MTT assy. Briefly, LCSCs were seeded at 5×10^3^ cells/well in a 96-well plate, pre-incubated for 24 h, then incubated with Fe_3_O_4_@SiNPs (free HSPI), HSPI or HSPI-loaded Fe_3_O_4_@SiNPs (HSPI&Fe_3_O_4_@SiNPs) for 24 h at concentrations ranging from 10 to 500 μg/mL, and then 10 μL MTT was added. After 4 h incubation, the formzan crystals were dissoloved in 150 mL DMSO and absorbance was measured at 570 nm with a reference wavelength of 630 nm.

*Multifunctional Nanoparticles Uptake by LCSCs*: LCSCs (1×10^4^ cells/well) were cultured on coverslip and incubated overnight at 37 ^o^C, then treated with 100 μg/mL PE-CD20 labeled Fe_3_O_4_@SiNPs (CD20-Fe_3_O_4_@SiNPs) and Fe_3_O_4_@SiNPs (encapsulated with PE dye) for 1 h. The uptake of NPs in cells was observed by co-localizing with lysosome tracker with a confocal microscope (SPE, Leica, Germany) after nuclear staining with DAPI.

*In Vitro Targeted Internalization:* LCSCs (1×10^4^ cells/well) were seeded in the 24-well plate and cultured overnight, then added 100 μg/mL CD20-Fe_3_O_4_@SiNPs and Fe_3_O_4_@SiNPs and incubated for 1 h. The cells were then fixed and stained for bio-TEM according our previous work 36, 37. The images were captured by TEM (FEI / Philips Tecnai 12 BioTWIN).

### *In Vitro* and* In Vivo* Combined Therapeutic Effects on LCSCs

*In vitro Thermotherapy and Chemotherapy by Applying AMF*: LCSCs (5 ×10^4^ cells/mL) were cultured in the 6-well plate for 24 h. Then, cells were separately treated with 100 μg/mL Fe_3_O_4_@SiNPs, HSPI&Fe_3_O_4_@SiNPs, SiNPs, HSPI, CD20-Fe_3_O_4_@SiNPs, and CD20-HSPI&Fe_3_O_4_@SiNPs for 1 h. Following two washes with PBS, cells were placed inside the AMF (5 cm diameter 10-turn induction coil powered by a 5 kW, 350 kHz) and heated to a defined temperature (between 37 and 50 ^o^C) for 30 min. The temperature of cell solution was monitored by a thermometer. The traditional heating method (water bath heater) was used to compare with AMF heating. The viability of cells was evaluated by MTT assay.

*Flow Cytometry Analysis*: The apoptosis and necrosis of LCSCs (treated with CD20-HSPI&Fe_3_O_4_@SiNPs under AMF) were tested by Apoptosis Detection Kits (YO-PRO-1/7-AAD, Invitrogen) according to the manufacturer's protocol, and then analyzed by BD FACSCanto II flow cytometry (BD Biosciences).

*Human Lung Cancer Xenograft*: BALB/c nude mice with 5 weeks old and 16-18 g weight were provided from Queen Elizabeth Hospital (Hong Kong, China). All the animal experiments were performed incompliance with the guidelines outlined in the Guide for the Care and Use of Laboratory Animals of Hong Kong. Tumor bearing mouse mode was established by subcutaneous injection of LCSCs (3×10^4^ cells/200 µL) into back region. Tumor growth in each mouse was closely observed every 4 days. The formula of length × width × depth × π/6 was used to calculate the tumor volume.

*In Vivo Distribution of MNPs*: CD20-HSPI&Fe_3_O_4_@SiNPs or HSPI&Fe_3_O_4_@SiNPs were injected into the LCSCs bearing mice* via* the retro-orbital sinus. Images were captured at 0.5, 1, 2, and 24 h by using the *in vivo* imaging system (Xenogen IVIS^®^ Spectrum). The* ex vivo* image of organs including tumor, kidneys, liver, lung, heart, and spleen were taken after sacrificing the mice. Furthermore, the content of Fe element in organs was tested to investigate the distribution of NPs by the inductively coupled plasma mass spectrometry (ICP-MS, Thermo Scientific™ ELEMENT 2™).

*Efficacy of Combination Thermotherapy and Chemotherapy in Animal Models*: The tumor bearing mice (tumor volume about 100 mm^3^) were randomly divided into five groups (n=10): CD20-Fe_3_O_4_@SiNPs, CD20-HSPI&Fe_3_O_4_@SiNPs, HSPI&Fe_3_O_4_@SiNPs, CD20-HSPI@SiNPs, and PBS. The samples (50 mg/kg) were injected to nude mice *via* the retro-orbital sinus once a week. After one day injection, the mice were then exposed to AMF (induction coil: 10 cm diameter and 12-turn; power: 5 kW) for 30 minutes (3 times each week). The tumor volume and body weight were measured every 4 days.

*Metastatic Model of LCSCs*: LCSCs (1×10^4^ cells/100 µL) were injected into the tail vein of five to six-week-old BALB/c nude mice. The mice were sacrificed after 14 days injection, and the organs were excised and fixed in a formaldehyde neutral buffer solution. Nodules, in the organs, were enumerated with the aid of a microscope. The tumor in organs was then analyzed by immunohistochemisical (IHC) staining.

*In Vivo Efficacy of Combination Thermotherapy and Chemotherapy on Metastasis*: After the establishment of metastatic model, the metastasis study was carried out. The LCSCs-injected mice were randomly assigned to four groups (n=3). The mice were then administrated with saline, HSPI&Fe_3_O_4_@SiNPs, and CD20-HSPI&Fe_3_O_4_@SiNPs at concentration of 50 mg/kg *via* the retro-orbital sinus once a week. After one day injection, the mice were then exposed to AMF (induction coil: 10 cm diameter and 12-turn; power: 5 kW) for 30 min (2 times each week). After 4 weeks, the mice were sacrificed and immediately the lung was collected to count the nodules.

### Biosafety Evaluation

*Hemolysis Assay and Biochemical Analysis*: The whole blood was centrifuged at 3000 rpm for 5 min to harvest the red blood cells (RBCs). The hemolytic effect of NPs was evaluated by incubating with 50 μL CD20-HSPI&Fe_3_O_4_@SiNPs (final concentration 1 mg/mL) at 37^o^C for 1 h. The supernatant absorbance was tested at 540 nm by microplate reader.

*Immune Cell Analysis*: To further investigate the side effects of NPs on immune system of mice, the whole blood was collected into anticoagulant from NPs treated mice on day 1, 2, 3, 4, 5, 6, 7, and 40 post-injection. White blood cell populations were gated into lymphocytes, monocytes and macrophages, and neutrophils by using forward and side scatter analysis in a flow cytometry. Number of B-Cell from lymphocytes was then analyzed with antibodies against typical B-cell antigens (CD20). Mice without NPs injection were used as control.

*In Vivo Uptake of NPs in Bone Marrow-derived Mesenchymal Stem Cells (MSCs)*: MSCs were isolated from NPs treated mice on day 40 post-injection according to previous work 36, 37. The uptake of NPs in MSCs was analyzed by using a FACSCalibur flow cytometry system. Mice without NPs injection were used as control.

*Tumor Xenograft and Organ Tissues Staining*: Tumors and organs were collected for studying the therapeutic effects and side effects of MNPs on mice by IHC analysis. The IHC images were taken under a microscope (CX41, Olympus).

All the detailed information was shown in Supplementary Information.

### Statistical Analysis

All the experiments were performed as three independent trials, and the data were expressed as mean ± standard deviation (SD). Student's t-test or one way-ANOVA was used to determine the significant differences (p < 0.05).

## Results

### Lung Cancer Stem Cells (LCSCs)

In this study, LCSCs were isolated from the parental population of human lung tumor cells and characterized by immunofluorescence imaging for selected surface markers and stemness markers, such as CD20, CD15, ABCG2, and Oct4 (Figure S1). CD20-positive cells were sorted by flow cytometry and were determined to have stronger capacities of tumor sphere formation, migration, and invasion than CD20-negative cells. An *in vivo* tumorigenic study showed that the tumor formation of LCSCs was faster and resulted in an increased tumor burden compared with that observed for non-CSCs at the same cell number, indicating the high tumor-initiating capacity of LCSCs (Figure S2A-D). As these cells are highly tumorigenic, we hypothesized that LCSCs-targeted therapy may be the key to thoroughly eradicate lung tumor originated from cancer stem cells.

### Synthesis and Characterization of MNPs

**Figure 2:**
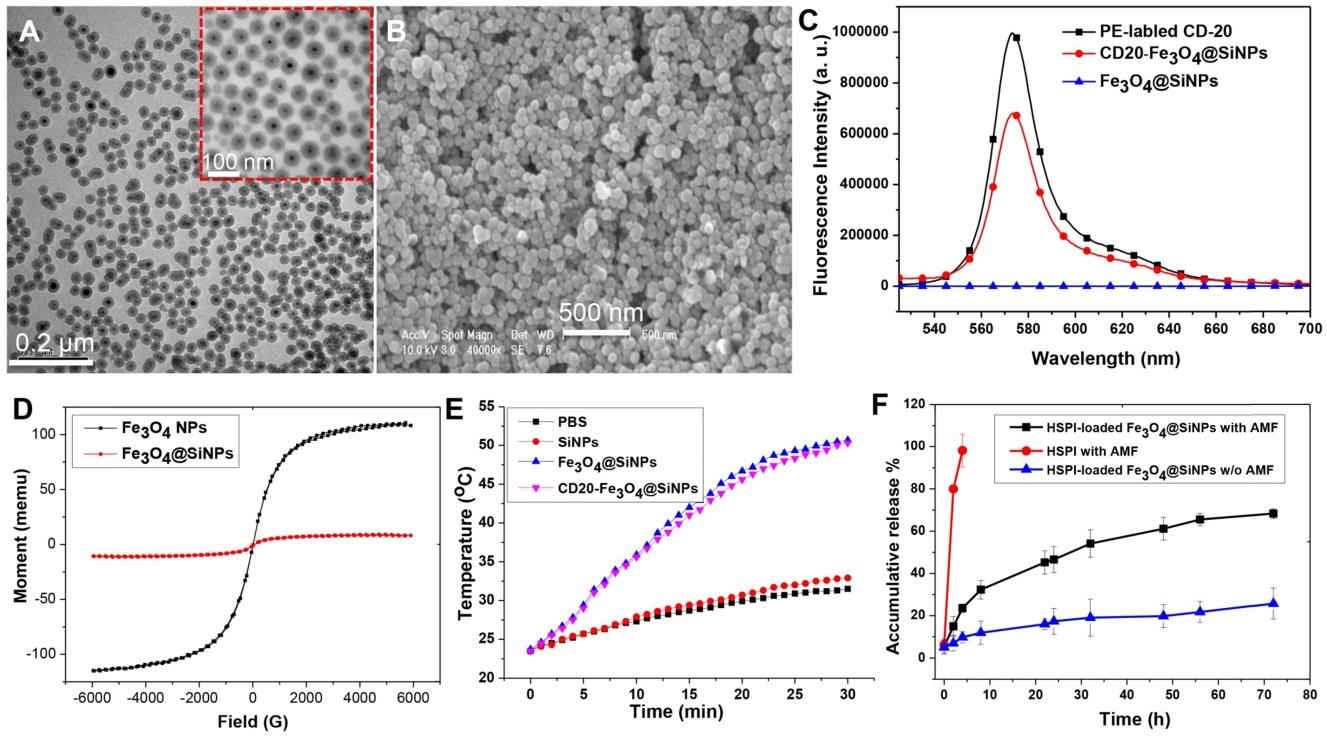
Characterization of MNPs. (A) The core/shell structure of MNPs is shown in TEM image and enlarged image. (B) SEM of the MNPs. (C) Fluorescent spectra of CD20-Fe_3_O_4_@SiNPs. (D) Magnetic hysteresis loops of Fe_3_O_4_@SiNPs and Fe_3_O_4_ NPs. (E) The increased temperature of PBS, SiNPs, Fe_3_O_4_@SiNPs, and CD20-Fe_3_O_4_@SiNPs under AMF. (F) *In vitro* drug release of HSPI/Fe_3_O_4_@SiNPs under AMF. Data are performed three times as the mean ± SD.

Transmission electron microscope (TEM) and scanning electron microscope (SEM) images demonstrated that the Fe_3_O_4_@SiNPs and CD20-Fe_3_O_4_@SiNPs were mono-dispersed in PBS buffer for a few weeks without aggregation. The sizes of MNPs were narrowly distributed about 55 ± 10 nm (Figure 2A and B). The sizes of Fe_3_O_4_@SiNPs were around 50 ± 10 nm (Figure S3A). Conjugation with the PE-CD20 antibody slightly changed the particle sizes (Figure S3B). As shown in Figure 2A, the Fe_3_O_4_ NP core (dark colour) was approximately 20 ± 5 nm and the thickness of silica shell was around 30 ± 5 nm. The surface charges and zeta potential of the Fe_3_O_4_@SiNPs and CD20-Fe_3_O_4_@SiNPs were -42.86 ± 3.74 and -22.04 ± 1.07 mV, respectively (Figure S3C). Figure S3D and S3E showed the collapsed structure of CD20-Fe_3_O_4_@SiNPs after AMF treatment. Furthermore, the conjugation of anti-CD20/PE on the surface of HSPI&Fe_3_O_4_@SiNPs was confirmed by fluorescent microscope (FluoroMax-4) that PE-CD20-labelled NPs was located at the same wavelength (580 nm) with free anti-CD20/PE (Figure 2C).

### Magnetic Hyperthermia Effect and Controlled Drug Release

Hysteresis curves obtained from a vibrating sample magnetometer (VSM) indicated the magnetization saturation (*Ms*) for the Fe_3_O_4_ NPs and CD20-Fe_3_O_4_@SiNPs. As demonstrated in Figure 2D, the curve through the origin suggested that both the Fe_3_O_4_ NPs and CD20-Fe_3_O_4_@SiNPs were superparamagnetic with the *Ms* values of 26 emu/g and 2.6 emu/g, respectively. The weaker magnetization of CD20-Fe_3_O_4_@SiNPs than naked Fe_3_O_4_@SiNPs is ascribed to the amount of magnetic materials in the sample (Figure S4).

A high *Ms* value is desirable to enhance the heating rate of the NPs under an AMF. As described in Figure 2E, the temperature of NP suspensions was increased over time under AMF. The highest temperature of Fe_3_O_4_@SiNP and CD20-Fe_3_O_4_@SiNP suspension respectively reached to 50.7 and 50.2 ^o^C, respectively, which is much higher than those generated from SiNP suspension and PBS solution. Therefore, with an even dispersion of the NPs in a neutral medium and effective heating, CD20-Fe_3_O_4_@SiNP can serve as a deliver system is a strong candidate for heat-triggered drug delivery and magnetic field induced thermotherapy. Furthermore, the specific absorption rate (*SAR*) was calculated according to the formula:


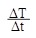

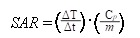


where C*_p_* is the heat capacity of water, and is the slope of the linear part of the heating curve, *m* is the concentration of magnetic NPs. The *SAR* of Fe_3_O_4_@SiNPs and CD20-Fe_3_O_4_@SiNPs were 75.4 W/g and 69.7 W/g, respectively, at 21 kA/m field strength and 350 kHz, indicating a good heating generation property and promising biomedical applications.

Nanoencapsulation efficiency plays a very crucial role in nano-based drug delivery systems. According to the formula and the standard curve of drug loading in Figure S5C, the EE was found to be 79.1 ± 2.5%, and LE was found to be 16.51 ± 3.3%. Controlled and sustained drug release is very important for drug delivery systems. The structure and encapsulation efficiency of the anticancer drug HSPI were shown in Figure S5. Figure 2F depicts the accumulative release profile of HSPI from the 1 mg/mL Fe_3_O_4_@SiNPs. A sustained release of HSPI from MNPs was obtained for up to 72 h (70% release) under an AMF. However, a drug release rate of only 21.5% was observed for up to 72 h without the AMF treatment, suggesting the possibility for controlled release *in vivo* via magnetically induced hyperthermia.

### Cellular Uptake and Internalization in LCSCs

**Figure 3:**
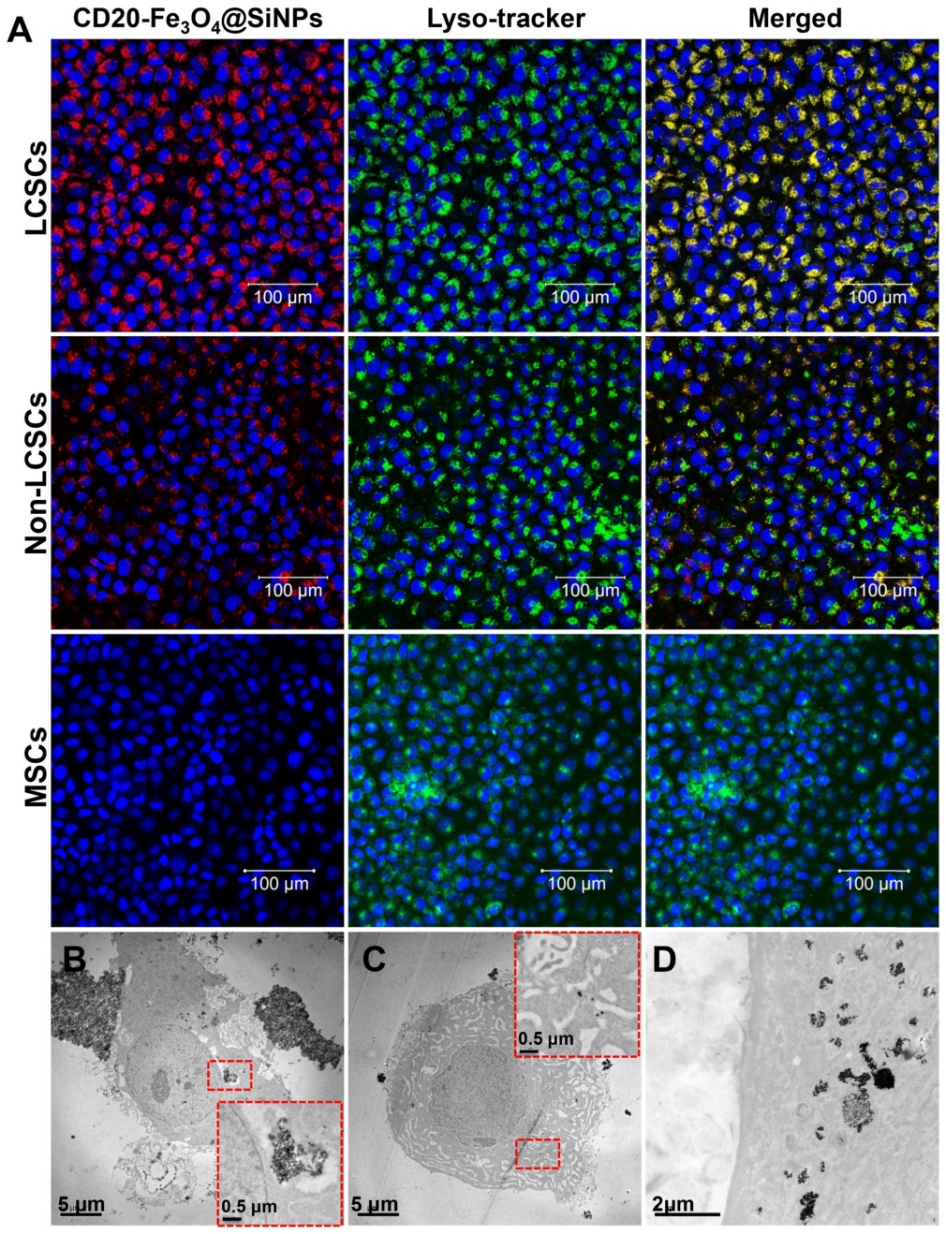
*In vitro* cellular uptake and internalization of CD20-Fe_3_O_4_@SiNPs by LCSCs, non-LCSCs, and MSCs. (A) Confocal images of cells treated with CD20-Fe_3_O_4_@SiNPs for 1 h. TEM images showed the internalization of (B) CD20-Fe_3_O_4_@SiNPs, (C) Fe_3_O_4_@SiNPs by LCSCs. (D) Subcellular localization of CD20-Fe_3_O_4_@SiNPs in LCSCs.

The cellular uptake of Fe_3_O_4_@SiNPs and CD20-Fe_3_O_4_@SiNPs by LCSCs (highly expressing CD20), differentiated LCSCs (low expressing CD20) and MSCs (non-expressing CD20) was investigated using laser confocal scanning microscopy. LCSCs (3^rd^ passage) were incubated with Fe_3_O_4_@SiNPs, CD20-Fe_3_O_4_@SiNPs, and anti-CD20 at 37 °C for 1 h at a concentration at 100 μg/mL. As seen in Figure S11, the specific targeting signal was observed after treatment with CD20-Fe_3_O_4_@SiNPs, and the signal of anti-CD20 was shown on the surface of LCSCs, indicating the receptor-mediated endocytosis. Moreover, Figure 3A demonstrates that the uptake of CD20-Fe_3_O_4_@SiNPs by LCSCs was higher than differentiated LCSCs (non-LCSCs) after 1 h incubation due to the decreased CD20 expression in differentiated LCSCs. Almost no signal was observed in CD20 negative MSCs after 1 h treatment. These results indicated the specific targeting of CD20-Fe_3_O_4_@SiNPs to CD20 positive cells. The conjugated anti-CD20 helped the MNPs enter the LCSCs with highly expressed CD20 more quickly *via* the receptor-mediated endocytosis pathway. The location of CD20-Fe_3_O_4_@SiNPs in cells was observed by co-localizing with the lysosome marker. Most of the CD20-Fe_3_O_4_@SiNPs entered the lysosomes after 1 h incubation.

Based on the results of the cellular uptake by LCSCs, the internalization of NPs was further studied by bio-TEM. Figure 3B showed that the CD20-Fe_3_O_4_@SiNPs were firstly aggregated near the cell membrane and then internalized after 1 h of incubation localizing in the lysosomes and cytoplasm (Figure 3B inserted image and Figure 3D enlarged image ). On the other hand, fewer Fe_3_O_4_@SiNPs were localized in the lysosomes or cytoplasm after 1 h of incubation, indicating that anti-CD20 facilitated the targeted receptor internalization (Figure 3C).

### *In Vitro* Thermotherapeutic and Chemotherapeutic Effects of MNPs on LCSCs

To evaluate the cytotoxicity of the NPs, LCSCs were incubated with free HSPI, Fe_3_O_4_@SiNPs, or CD20-HSPI&Fe_3_O_4_@SiNPs at concentrations of 10-500 μg/mL (corresponding HSPI concentrations were 5 nM to 1 μM) for 24 h, and then, the cell viability was evaluated by the MTT assay. Compared with free HSPI and Fe_3_O_4_@SiNP, the HSPI&Fe_3_O_4_@SiNP was more toxic to the LCSCs (Figure S6A). HSPI&Fe_3_O_4_@SiNPs showed a lower IC_50_ (100 nM) than free HSPI (1μM), which may be attributed to the improved targeting efficacy of the MNPs. In addition, the cytotoxicity of the Fe_3_O_4_@SiNPs was evaluated to determine the cytotoxicity caused by Fe_3_O_4_@SiNPs themselves (Figure S6B). The results indicated that Fe_3_O_4_@SiNPs had no cytotoxicity to LCSCs at concentrations of 10 to 200 μg/mL. Hence, the lower IC_50_ of the HSPI&Fe_3_O_4_@SiNPs was not due to the toxic effect of Fe_3_O_4_@SiNPs but was primarily due to the enhanced cellular internalization of HSPI with Fe_3_O_4_@SiNPs.

**Figure 4:**
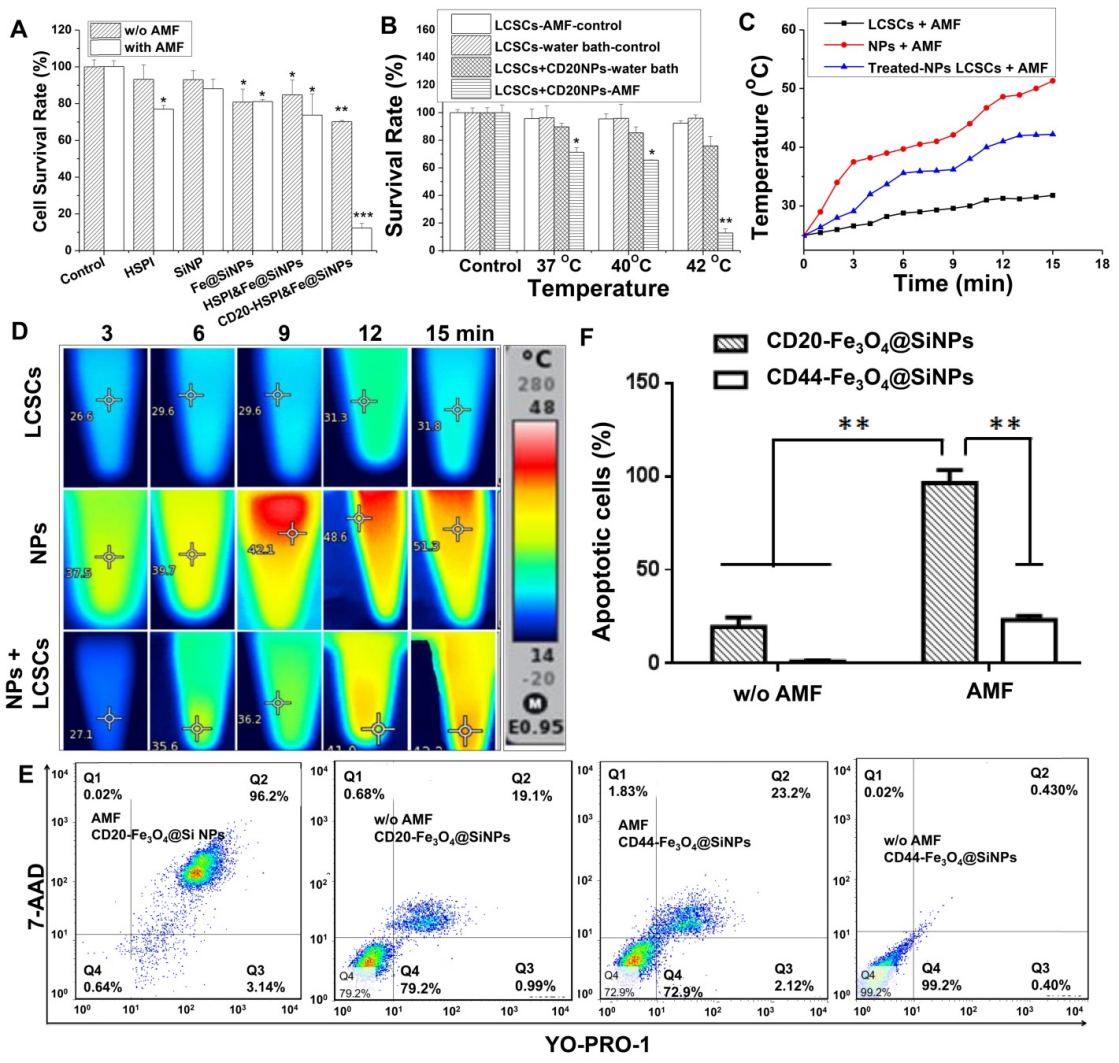
Effects of hyperthermia and chemotoxicity on LCSCs by applying AMF. (A) The MNPs induced a synergistic effect on LCSCs when compared with different components of the MNPs. (B) CD20-conjugated MNPs significantly suppressed LCSC growth under an AMF compared with heat treatment in a water bath at 37 °C, 40 °C or 42 °C. (C) The curve of increased temperature of LCSCs, MNPs, and LCSCs treated with MNPs. (D) Thermal images of LCSCs, MNPs, and LCSCs treated with MNPs. (E) The dot plots of LCSCs show the apoptosis and necrosis after treatment. (F) Statistical analysis of MNP-treated cells under an AMF and in a 42°C water bath.

To test the combinatorial thermotherapeutic and chemotherapeutic effects of CD20-HSPI&Fe_3_O_4_@SiNPs, LCSCs were incubated with MNPs and heated at 37 °C for 30 min by applying an AMF. Notably, the survival rate of LCSCs treated with MNPs was dramatically decreased compared to the control group (Figure 4A). Furthermore, the survival rate of LCSCs in the presence of HSPI, SiNPs, Fe_3_O_4_@SiNPs, and HSPI&Fe_3_O_4_@SiNP decreased to 77%, 88%, 81%, and 73% under the AMF, respectively (Figure 4B). As shown in Figure 4C and 4D, the temperature of the medium treated with CD20-HSPI&Fe_3_O_4_@SiNPs reached to 42^o^C, which is sufficient to kill the LCSCs. These results demonstrate the highly selective anti-tumor efficacy of combined CD20-HSPI&Fe_3_O_4_@SiNP thermo- and chemo-therapy triggered by AMF.

The cell death mechanism caused by the combined thermo- and chemo-therapy was evaluated by 7-AAD permeability (membrane integrity marker) and YO-PRO1 labelling (apoptotic marker). The medium containing LCSCs were heated by either a water bath or an AMF to 37 ^o^C for 30 min. Consistent with the above findings, the heating from water bath did not lead to notable cell death. 7-AAD-and YO-PRO1-positive cells were not observed after the heating process in the water bath (Figure 4E and 4F). By contrast, the percentage of LCSCs that were positive for both 7-AAD and YO-PRO1 after treatment with CD20-HSPI&Fe_3_O_4_@SiNPs reached 83.9%. This suggested the temperature at the LCSC surface was much greater than 37 ^o^C. However, there were no apoptotic cells (YO-PRO1-positive, 7-AAD-negative) after AMF treatment, suggesting the predominant form of cell death was necrosis.

### *In Vivo* Tumor-targeted Accumulation and Whole Body Distribution

**Figure 5:**
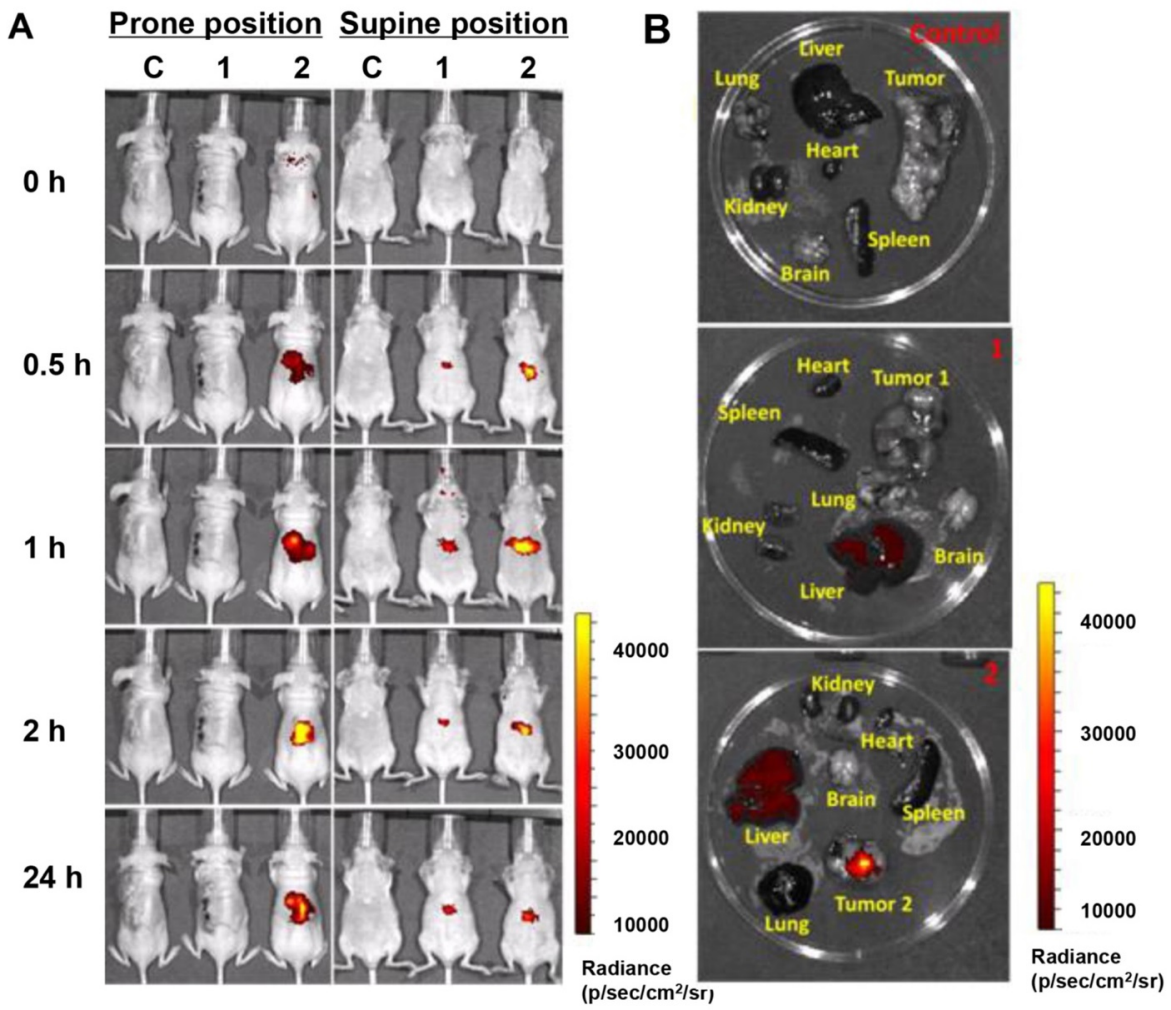
Tumor targeting ability and whole body distribution of MNPs. (A) Mice *in vivo* images after treatment with CD20-HSPI&Fe_3_O_4_@SiNPs and HSPI&Fe_3_O_4_@SiNPs for 0.5, 1, 2, and 24 h (intravenous injection). Most HSPI&Fe_3_O_4_@SiNPs gathered at the liver, whereas, the CD20-HSPI&Fe_3_O_4_@SiNPswere primarily concentrated in the tumor region. (B) *Ex vivo* images of mice after 24 h of treatment with CD20-HSPI&Fe_3_O_4_@SiNPs and HSPI&Fe_3_O_4_@SiNPs. (C: control; 1: HSPI&Fe_3_O_4_@SiNP injection; 2: CD20-HSPI&Fe_3_O_4_@SiNP injection)

The tumor-targeting efficacy and whole body distribution of CD20-HSPI&Fe_3_O_4_@SiNPs in tumor-bearing mice were then investigated using an *in vivo* imaging system. Figure 5A shows that the fluorescence signals of CD20-HSPI&Fe_3_O_4_@SiNPs and HSPI&Fe_3_O_4_@SiNPs (encapsulated with PE dye) were mostly located in the liver 30 min after injection. As time elapsed, the fluorescent signal was observed at the tumor site in CD20-HSPI&Fe_3_O_4_@SiNP-treated mice. At 24 h post-injection, the CD20-HSPI&Fe_3_O_4_@SiNP fluorescence signals were primarily located around the tumor with little fluorescence observed in the liver. However, there was no detectable signal in tumor site after treated with HSPI&Fe_3_O_4_@SiNPs, indicating the targeting efficiency of the anti-CD20 coating on the MNPs. The *ex vivo* imaging for the organs further confirmed the specific targeting efficiency and tumor accumulation of the CD20-HSPI&Fe_3_O_4_@SiNPs (Figure 5B). Only a weak signal was obtained in the liver after 24 h, with no obvious fluorescent in the spleen, lung, heart, or kidney, indicating that unbound MNPs were excreted by metabolism.

### *In Vivo* MNPs Showed Good Biocompatibility

**Figure 6:**
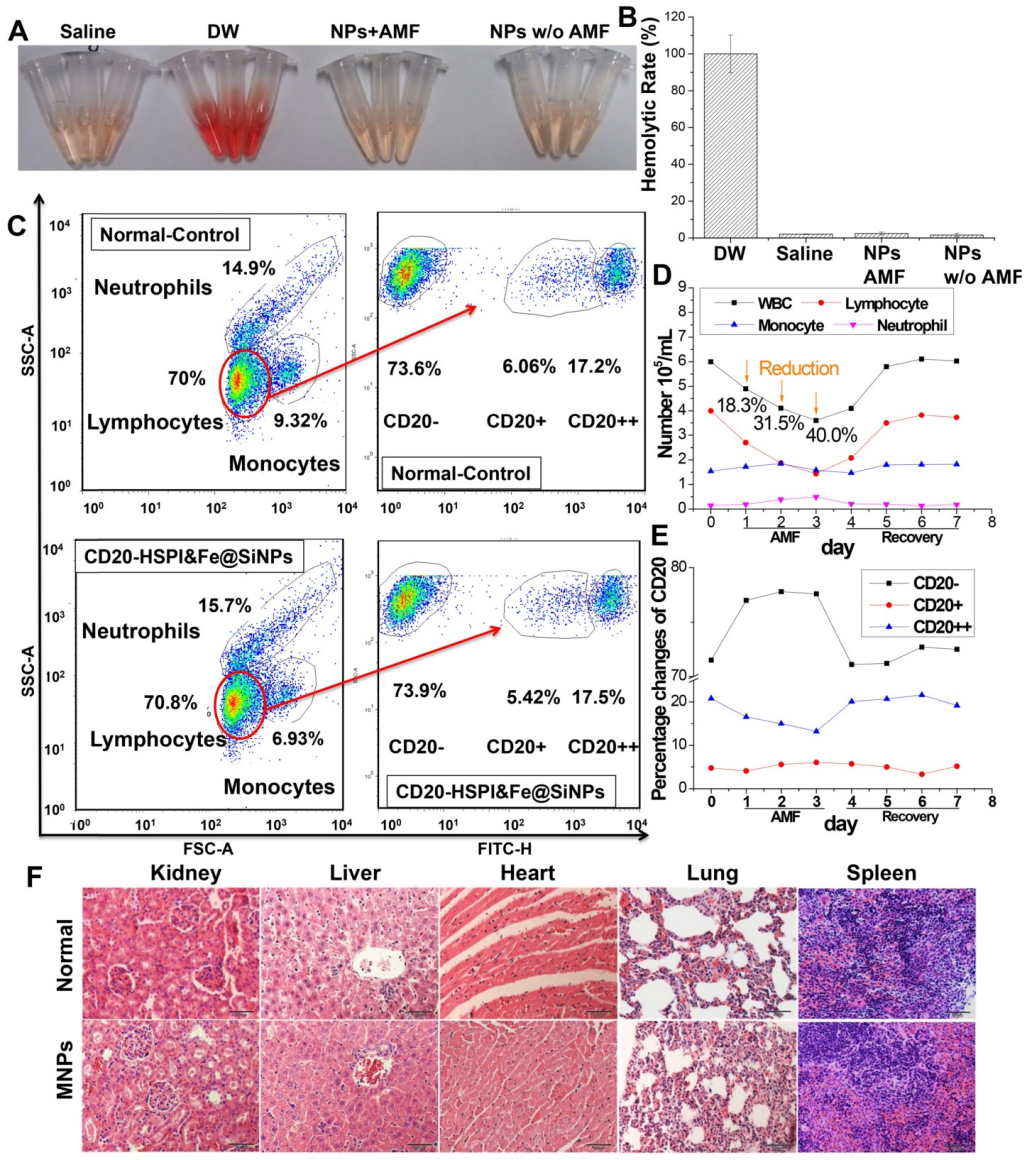
Biocompatibility of MNPs *in vivo*. (A) Haemolysis analysis of MNP treatment under an AMF. (B) Statistical analysis of haemolysis. (C) WBC counts and B-cell changes in mice after CD20-HSPI&Fe_3_O_4_@SiNP-mediated AMF treatment. Percentage of (D) WBCs and (E) B-cells in mice with CD20-HSPI&Fe_3_O_4_@SiNPs after 7 days of recovery. (F) Histological images revealed no signs of MNP-induced toxicity after 36 days. No anomalies were observed in the organs. The images were taken at 20× magnification. The data represent 3 separate experiments.

Before evaluating the tumor targeting and therapeutic efficacy in mice, the blood compatibility of CD20-HSPI&Fe_3_O_4_@SiNPs was evaluated by haemolysis assay and whole blood analysis. For the haemolysis analysis, if erythrocytes are lysed, haemoglobin will be released and the supernatant will appear red, resulting in absorbance at 540 nm. As shown in Figure 6A and 6B, there was no haemoglobin of RBCs after treatment of 1 mg/mL MNPs, indicating a good haemocompatibility (< 4% haemolysis). To evaluate the effects of MNPs on white blood cells, mice were injected with NPs and treated under AMF for 30 min. White blood cell populations were gated into lymphocytes, monocytes, and neutrophils using forward and side scatter analysis in a flow cytometer. As seen in Figure S9A, no significant difference in immune cell numbers between the NP-treated and control groups. Besides, the functions of liver and kidney were also evaluated, including renal functions (Cr and UA) and liver functions (AST, ALT, and ALP). As shown in Table S1, there was no significant difference between control and MNPs groups. These results demonstrate that the MNPs, with their good blood compatibility, can be used for *in vivo* experiments.

Immune cell injury and recovery induced by CD20-HSPI&Fe_3_O_4_@SiNP treatment were assessed according to white blood cell (WBC) counts, including lymphocytes, monocytes, and neutrophils (Figure 6C-E). Lymphocyte levels in the WBC population were significantly reduced 3 days after AMF treatment, and the levels returned to normal by Day 6. In addition, a detailed B-cell analysis was performed using the CD20 antibody. Although the B-cell nadir on Day 3 was significantly reduced by treatment with CD20-HSPI&Fe_3_O_4_@SiNPs, a faster recovery of B-cell counts after Day 4 was observed, and the counts returned to basal levels as early as Day 6 (Figure 6E). It is noteworthy that the number of B-cells began to increase at approximately Day 4 whereas recovery of WBCs was exhibited at Day 6. These results suggest that damaged B-cells begin to recover at approximately Day 4 after AMF treatment by activation of the haematopoietic function. Importantly, no CD20-HSPI@Fe_3_O_4_@SiNP uptake was observed in MSCs from the bone marrow of CD20-HSPI@Fe_3_O_4_@SiNP-treated mice (Figure S9B).

The* in vivo* toxicity of the MNPs was continuously evaluated over 36 days of AMF treatment. The histopathologic effect of NPs on various organs such as the heart, lung, liver and kidney was investigated. As shown in Figure 6F, compared to the control group, the treated groups showed no obvious histopathologic changes. Furthermore, no NPs accumulation was observed in the tissues. Thus, the histopathological analysis confirms that the CD20-HSPI&Fe_3_O_4_@SiNPs did not accumulate non-specifically in the organs and did not damage the organs by over-accumulation.

### *In Vivo* Combined Thermo- and Chemotherapy Inhibited Tumor Growth

To determine the efficacy of CD20-HSPI&Fe_3_O_4_@SiNPs in combined anti-tumor thermotherapy and chemotherapy, the LCSCs xenograft was established by injecting LCSCs to the back of nude mice, and then separated into several experimental groups (n = 10) after the tumor volume reach to 100 mm^3^. Since the LCSCs model is a high-degree malignancy model, and the tumor volume increased to approximately 1500 mm^3^ within 14 days. A total tumor volume of more than 2000 mm^3^ was deemed moribund or fatal by a veterinary consultation.

**Figure 7:**
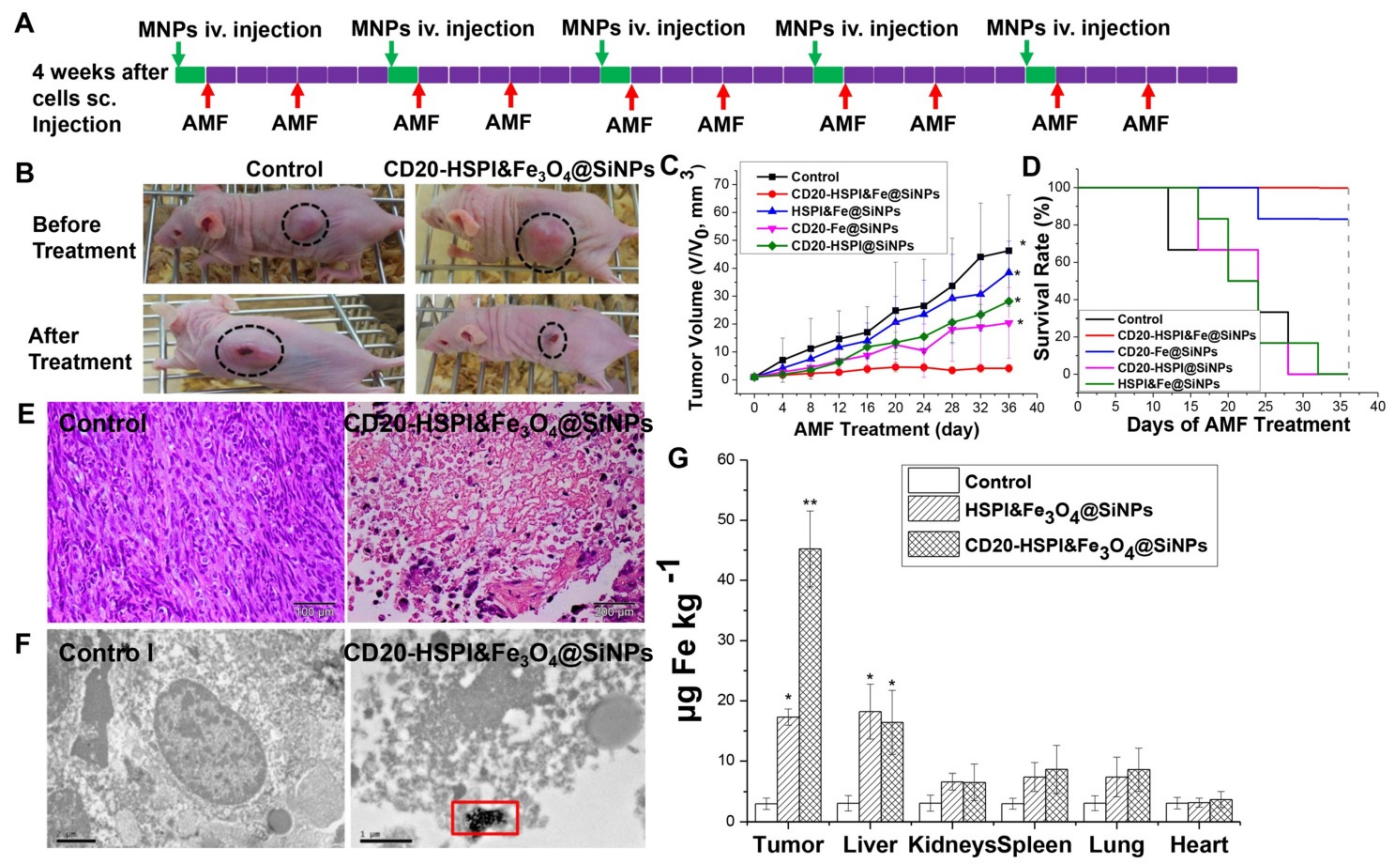
*In vivo* inhibition of tumor growth by administration of MNPs with combind thermo- and chemo-therapy. (A) Nude mice treatment scheme. (B) Tumor-bearing mice images before AMF treatment and 36 days after AMF treatment. (C) The curve of tumor volume (V/V_initial_) after MNPs-mediated AMF treatment. (D) Cumulative survival rate of nude mice injected with NPs. Subcutaneous tumors after injection with saline, HSPI&Fe_3_O_4_@SiNPs and CD20-HSPI&Fe_3_O_4_@SiNPs. (E) H&E-stained tumor tissue sections of control and CD20-HSPI&Fe_3_O_4_@SiNP-treated mice at 36 days after AMF treatment. (F) TEM images of tumor tissue in mice treated with saline and CD20-HSPI&Fe_3_O_4_@SiNPs by *i.v.* injection. Nanoparticles accumulated in the tumor tissue, which was seriously damaged after 36 days of AMF treatment. (G) Fe element content in organs showed the biodistribution of the MNPs in mouse after 24 h *i.v.* injection. Experiments were performed three times (n = 10).

CD20-HSPI&Fe_3_O_4_@SiNPs dispersed in normal PBS were injected into the tumor-bearing mice by the retro-orbital sinus. The mouse was then received the combined treatment under an AMF for 30 min at 350 kHZ, and the tumor volume was observed every 4 days up to 36 days. As shown in Figure 7A-C, the control group showed faster tumor growth than the group that received the combined thermo- and chemo-therapy with CD20-HSPI&Fe_3_O_4_@SiNPs. In the treatment group, the tumor growth was dramatically inhibited, with almost no apparent growth. In comparison, no significant effect was observed in the group that treated with unmodified HSPI&Fe_3_O_4_@SiNPs. During the observation period, the body weight of mice in each group was increased proportionately (Figure S7A). As shown in Figure 7C, treatment with CD20-HSPI&Fe_3_O_4_@SiNPs effectively inhibited tumor growth, and the mean survival period of mice treated with CD20-HSPI&Fe_3_O_4_@SiNPs was extended to 36 days from 12 days for the control groups (Figure 7D). The mice treated with PBS had the lowest body weights of all the groups (Figure S7A).

To further evaluate the anti-cancer efficiency of MNPs, *ex vivo* histology study of the tumor tissue was performed. The tumor tissue of the control group was found to be relatively well maintained with cancer nests. However, plenty of necrotic cells (with a dense purple nucleus and dark eosinophilic cytoplasm) were observed, indicating the significant necrosis of tumor tissue after MNPs treatment (Figure 7E). To better determine the therapeutic efficacy of CD20-HSPI&Fe_3_O_4_@SiNPs, tumor specimens (after 36 days of AMF treatment) were stained with anti-CD20/PE for immunohistochemical analysis. The decreased expression of CD20 in the tissue indicated the depletion of LCSCs in tumor with CD20-HSPI&Fe_3_O_4_@SiNPs treatment (Figure S7B). Additionally, nanoparticle accumulation was observed in the tumor tissues by bio-TEM imaging, indicating the tumor-targeting capacity of the MNPs (Figure 7F). Data obtained for the Fe content of organs from the mouse are individually shown in Figure 7G, together with the controls (white columns). Elevated Fe levels were found in tumor under study for the NPs-treated mouse. These results unequivocally demonstrate the destruction of the tumor tissue due to the heat generation from Fe_3_O_4_ NPs under an AMF. To investigate the long-term accumulation of NPs in the metabolic organs, the Fe content in liver and kidney was analyzed with the increase of post-injection time. As shown in Figure S10, the high Fe content was observed in liver and kidney after 4 h injection. However, the Fe level in liver and kidney was dramatically decreased to the negligible content after 24 h with the whole body blood circulation and metabolism.

### Systematic Administration of MNPs in LCSC-Induced Lung Metastasis Mice

**Figure 8:**
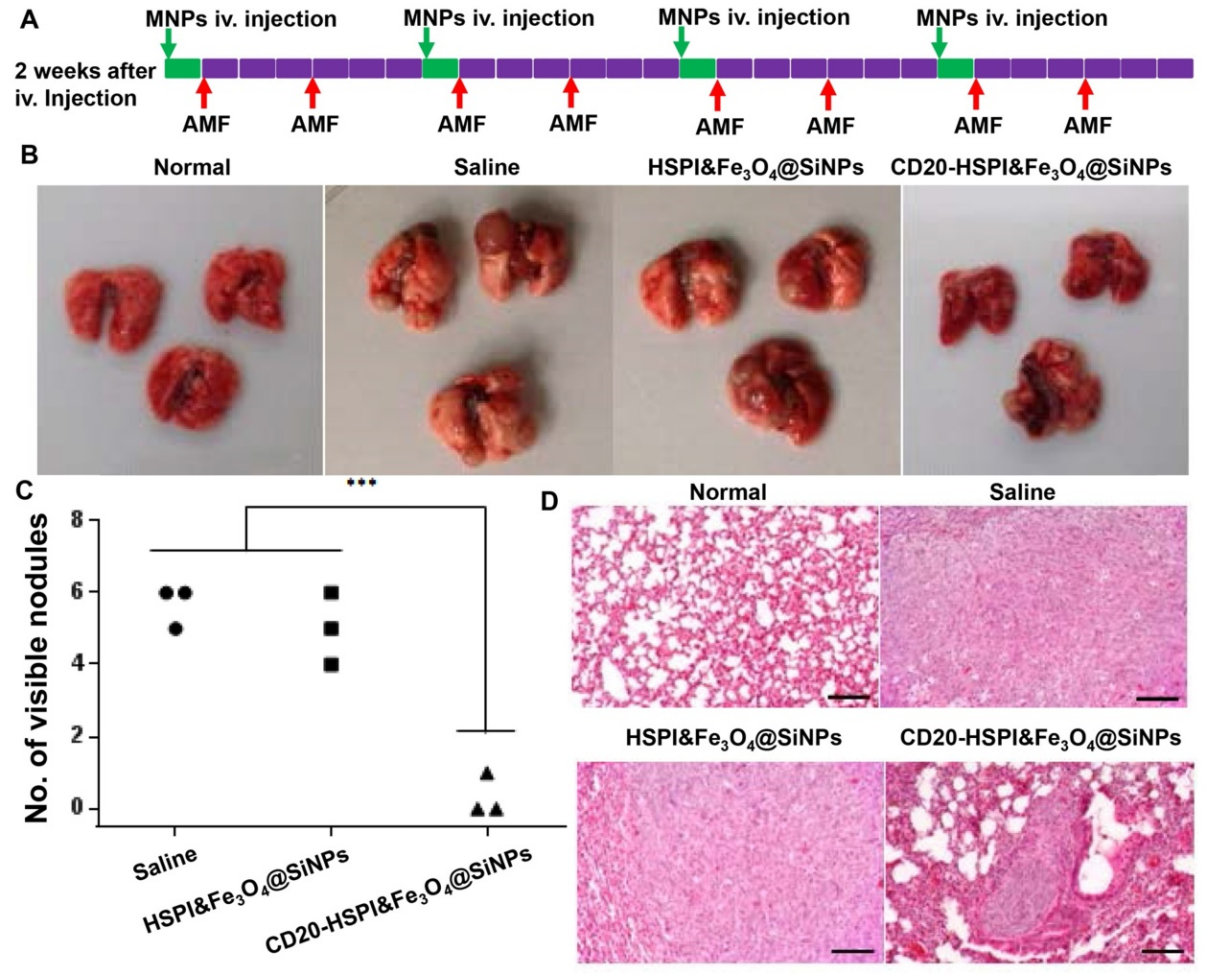
Systematic administration of MNPs in LCSC-established lung metastasis mice. (A) Treatment scheme of MNPs in a LCSC lung metastasis mouse model. (B) Macroscopic view of mouse lungs with different treatments: control, metastasis lung treated with saline, HSPI&Fe_3_O_4_@SiNPs and CD20-HSPI&Fe_3_O_4_@SiNPs. Lung nodules were observed on the surface. (C) Statistical analysis of mouse lung nodules in the different treatment groups. (D) H&E-stained mouse lung tissue sections from the control, metastasis lung treated with saline, HSPI&Fe_3_O_4_@SiNPs and CD20-HSPI&Fe_3_O_4_@SiNPs after AMF treatment.

To evaluate the efficacy of MNPs on LCSC-induced metastasis, LCSCs were intravenously injected into the mouse tail vein to establish a lung metastasis model. As shown in Figure S12, the tumors were found in lung, while, no tumor was observed in other organs. Lung is a frequent site of metastasis formation from both primary lung carcinomas and other extrapulmonary neoplasms due to their complex microenvironment, which generates pre-metastatic niches to support recruitment and colonization of metastatic cells 38, 39. The well-established LCSCs lung metastasis model was also utilized to evaluate the combined therapeutic efficacy of MNPs. After two weeks of injection, the mice were randomly separated into 3 groups with treatment of saline, HSPI&Fe_3_O_4_@SiNPs, and CD20-HSPI&Fe_3_O_4_@SiNPs. The treatment schedule for MNPs under an AMF was shown in Figure S8A. After 24 h of *i.v.* injection, the mice were subjected to an AMF for hyperthermia and chemotherapy treatment. The body weight of the mice was monitored and showed no significant change during the 4 weeks of treatment (Figure S8). The mice were then sacrificed to harvest the lungs. The nodules on the lung surface were counted. Statistical analysis showed that there were significantly fewer nodules in the mice treated with CD20-HSPI&Fe_3_O_4_@SiNPs than in mice treated with saline or HSPI&Fe_3_O_4_@SiNPs (Figure 8B and 8C). H&E staining showed that there was a decrease not only in nodule number but also in nodule size (Figure 8D).

## Discussion

Intratumoural heterogeneity represents a major obstacle to the development of effective cancer therapies. Ample evidence suggests that tumors may be driven by a very small part of cancer stem cells that have the ability to undergo self-renewal, develop resistance to conventional therapy, and differentiate into diverse cancer cell populations that constitute the bulk of the tumor 40-2.The recent identification of putative CSCs led to a quest for efficient cancer therapies. Although there are no universal markers for CSCs, many works have employed some surface antigens to identify the CSCs 43. Therefore, the development of CSC-targeted therapies offers a potential therapeutic approach for the complete elimination of CSCs and cancer cells to achieve a significantly better outcome for lung cancer patients.

Clinical results have showed that NP-based drug delivery systems improved the cancer therapeutic efficiency and reduced the side effects by achieving the tumor-targeted localization and cellular uptake, but CSCs-targeted cancer therapy by NP-based combined thermotherapy and chemotherapy has not been fully investigated. In this study, MNPs were designed and synthesized with magnetic cores and silica shell (for loading anticancer drugs). The surface of silica was conjugated with anti-CD20 against LCSCs for targeted and combined thermo- and chemo-therapy by applying an AMF. To investigate the magnetic and heat generation properties of CD20-Fe_3_O_4_@SiNPs, we measured the magnetization saturation and generated a hysteresis curve, indicating the superparamagnetic property of CD20-Fe_3_O_4_@SiNPs and AMF-induced heating ability (Figure 2E). The drug release analysis suggested that MNP complex enabled longer HSPI retention than bare HSPI *in vitro* in response to an AMF.

Recently, cancer treatments by nanopaticle-induced hyperthermia have been pursued using nanocomplexes with magnetic NPs. However, most of those nanocomplexes only targeted cancer cells but not CSCs, resulting in relapse of the tumor 44, 45. Moreover, the overexpression of heat shock proteins in cancer cells under thermal conductions triggers a defence mechanism to protect tumor cells from subsequent and more severe temperature challenges 46. This study utilized a heat shock protein inhibitor (Alvespimycin, 17-DMAG (phase II/III), Figure S5) that targets HSP90 pathways and is under investigation in FDA-sanctioned clinical trials. This inhibitor was encapsulated in the magnetic NPs as a chemotherapeutic agent for simultaneous thermotherapy and chemotherapy. Additionally, the MNPs exhibited targeted-delivery to LCSC upon modification with a CD20 antibody. The ability to target LCSCs using the CD20-HSPI&Fe_3_O_4_@SiNPs was further confirmed *in vivo* using a xenograft mouse tumor model. The uptake results showed that conjugation with the anti-CD20 facilitated the targeting of LCSCs after 1 h of incubation. However, the Fe_3_O_4_@SiNP uptake rate of LCSCs increased slightly with the time increasing, suggesting nonspecific uptake of NPs over long time incubation, consistent with other works 47. The specific targeting effect of CD20-Fe_3_O_4_@SiNPs to LCSCs was further demonstrated by bio-TEM. This study showed that NPs conjugated with anti-CD20 can facilitate the process of cellular internalization, and rapidly distributing in cytoplasm and lysosomes. The mechanism of uptake in CSCs was due to the interaction between CD20 receptor on the surface of LCSCs and anti-CD20, which induces the ubiquitination, leading to the endocytosis of MNPs 48, 49*.* The body distribution of MNPs clearly indicated that anti-CD20 can increase the tumor localization of NPs within a short time, as observed in many other studies 50-52. The *ex vivo* imaging also confirmed the tumor targeting *in vivo*, where the fluorescent signals of MNPs were clearly observed in the tumors. Little signal was observed in the liver after 24 h, indicating the quick clearance of NPs by the liver within 24 h after injection. MNPs signal was observed in other organs. The haemolysis analysis showed that the MNPs have good blood compatibility. After intravenous CD20-HSPI&Fe_3_O_4_@SiNP treatment, no changes were observed in lymphocyte, monocyte, macrophage, or neutrophil numbers relative to those in the control (Figure S9A). To evaluate the effects of MNPs on the normal stem cells, we incubated MSCs with NPs. The results showed that the uptake of the CD20-HSPI&Fe_3_O_4_@SiNPs was not detected in MSCs obtained from bone marrow and blood (Figure S9B), indicting specific targeting of the MNPs to CSCs. Due to the anti-CD20 on the surface of NPs, the potential deleterious side effects of non-targeted treatment was minimized. This specific LCSCs-targeting property demonstrates a significant advantage of nano drug delivery system for clinical applications. Another significant advantage of this MNP platform is the potential for combined thermotherapy and chemotherapy.

Thermotherapy, also called hyperthermia, is being rapidly developed as a method for treating cancer. Temperatures ranging from 40 ℃ to 50 ℃ generated from iron oxide NPs under an AMF is considered optimal for hyperthermia 53, 54. In the present work, we first appraised the thermotherapeutic effects of CD20-Fe_3_O_4_@SiNPs *in vitro*. Besides causing the expected LCSC death, the AMF-controlled CD20-Fe_3_O_4_@SiNP-mediated thermotherapy also induced the heat shock protein (HSP) expression, which is a stress-response protein and protects tumor cells from further damage. In addition, the hyperthermia can not only kill LCSCs but also normal cells when exposed to temperatures of 42-45 °C. To achieve the selective elimination of LCSCs at a lower temperature (37 °C), the HSP90 inhibitor 17-DMAG was encapsulated in CD20-Fe_3_O_4_@SiNPs to suppress the HSP90 expression and to overcome the thermal resistance of LCSCs. We next investigated both the thermotherapeutic and chemotherapeutic effects of CD20-HSPI&Fe_3_O_4_@SiNPs on the survival of LCSCs at 37 °C under an AMF for 30 min. The CD20-HSPI&Fe_3_O_4_@SiNPs specifically targeted to LCSCs and reduced the survival rate upon AMF application. Furthermore, the apoptotic and necrotic analysis by flow cytometry confirmed that the MNPs killed LCSCs by causing critical membrane damage and consequent necrotic cell death. The temperature in the LCSCs increased to above 42 °C, which caused cell membrane damage and consequent necrosis of cells, indicating that the cell death was mainly necrosis after MNPs-mediated AMF treatment.

Tumor growth may be effectively inhibited *in vivo* by selectively targeting CSCs with a combination of AMF-induced thermal destruction and chemotherapeutic drugs utilizing the multiple functions of NPs. Tumor-targeting efficacy of CD20-HSPI&Fe_3_O_4_@SiNPs was then evaluated in mice bearing tumors derived from human LCSCs. This study revealed that the tumor growth of LCSCs-xenograft mice was complete inhibited by the combined thermotherapy and chemotherapy, resulting in the elimination of LCSCs. The tumor growth of the group that received thermotherapeutic and chemotherapeutic treatment with CD20-HSPI&Fe_3_O_4_@SiNPs was inhibited under AMF treatment. For the control groups, the tumor size increased dramatically on the untreated mice and mice treated with HSPI&Fe_3_O_4_@SiNPs. Tumor tissue subjected to the combined treatment with CD20-HSPI&Fe_3_O_4_@SiNPs was analysed using H&E staining. The accumulation of MNPs in the tumor site caused the necrosis of LCSCs and cancer cells without causing damage on the surrounding normal tissue. Furthermore, the immunofluorescent staining results showed that no CD20 positive cells were observed in xenograft tumors, indicating the elimination of LCSCs in tumor by the specifically targeting to LCSCs and highly therapeutic efficacy of the CD20-HSPI&Fe_3_O_4_@SiNPs. Overall, these results confirmed the LCSC-targeting ability and the anti-tumor efficiency of the combined thermotherapeutic and chemotherapeutic nanodelivery system.

The MNPs have been proven to address many challenges in the scientific research field, such as diagnosis, drug delivery, and cancer treatment. The need to investigate toxicity *in vivo* is becoming increasingly important for their translation into clinical settings. In this study, the post-mortem histopathology of the lung, heart, kidneys, liver, and spleen was also analysed to evaluate any potential changes in organs in tumor-bearing mice. There were no obvious morphological differences between the tumor-bearing mice without treatment and the CD20-HSPI&Fe_3_O_4_@SiNP-treated mice. To comprehensively understand the response of immune cells and bone marrow to MNP-mediated AMF treatment, especially in cells that constitute the haematopoietic niche, peripheral blood and whole bone marrow (primarily composed of bone MSCs) were collected to evaluate changes in WBCs, especially B-cells. CD20 is a B-cell-specific differentiation antigen that is expressed on mature B-cells but not on early B-cell progenitors or later mature plasma cells 55. In this study, the B-cell nadir on Day 3 was significantly reduced by treatment with CD20-HSPI&Fe_3_O_4_@SiNPs, but new pre-B-cells were generated by differentiation of haematopoietic stem cells during the recovery period. Thus, the MNP-based nanodrug delivery system may offer a promising clinical platform for the combined thermotherapy and chemotherapy with good biocompatibility and targeting capability.

## Abbreviations

AMF: alternating magnetic field
ALP: alkaline phosphatase
ALT: alanine aminotransferase
AST: aspartate aminotransferase
CSCs: cancer stem cells
Cr: creatinine
DMSO: dimethyl sulfoxide
HSPI: heat shock protein inhibitor
ICP-MS: Inductively Coupled Plasma - Mass Spectrometry
IHC: immunohistochemistry
LCSCs: lung cancer stem cell
*Ms*: magnetization saturation
MSCs: mesenchymal stem cells
MNP: multifunctional nanoparticle
NP: nanoparticle
PBS: phosphate buffer saline
RBCs: red blood cells
SEM: scanning electron microscopy
SCLC: small cell lung cancer
TEOS: tetraethoxy orthosilicate
TEM: transmission electron microscopy
UA: uric acid
VSM: vibrating sample magnetometer
WBCs: white blood cells
MTT: 3-(4,5-dimethyl-2-thiazolyl)-2,5-diphenyl-2-H-tetrazolium bromide
