# Supplementary material for: Targeted destruction of cancer stem cells using multifunctional magnetic nanoparticles that enable combined hyperthermia and chemotherapy
Source: Theranostics. 2020 Jan 1;10(3):1181–96. doi: 10.7150/thno.38989 (PMC6956796; doi:10.7150/thno.38989)
Supplement: Supplementary file 1 — Supplementary figures and tables. [file thnov10p1181s1.pdf]

Supporting Information

Targeted destruction of cancer stem cells using multifunctional magnetic nanoparticles that enable combined hyperthermia and chemotherapy

Dandan Liu [a, b, c], Yingcai Hong [d], Yaping Li [a, b], Chong Hu [a, b], Tak-Chun Yip [e], Wai-Kin Yu [a, b], Yu Zhu [f], Chi-Chun Fong [a, d], Weimao Wang [a], Siu-Kie Au [e], Shubin, Wang [f], Mengsu Yang [a, b *]

a Department of Biomedical Sciences, City University of Hong Kong, Hong Kong, China;

b Key Laboratory of Biochip Technology, Biotech and Health Centre, Shenzhen Research Institute of City University of Hong Kong, Shenzhen 518057, China;

c College of Chemistry and Environmental Science, Key Laboratory of Medicinal Chemistry and Molecular Diagnosis of the Ministry of Education, Hebei University, Baoding 071002, China;

d Department of Thoracic Surgery, Shenzhen People's Hospital, the Second Clinical Medical College of Jinan University, Shenzhen 510000, China;

e Department of Clinical Oncology, Queen Elizabeth Hospital, Hong Kong, China;

f Department of Oncology, Peking University Shenzhen Hospital, Shenzhen 518036, China

* Corresponding author: Mengsu Yang

Tel.: (852) 3442-7797; Fax: (852) 3442-0552

* Email: bhmyang@cityu.edu.hk

Methods

Preparation of Heat Shock Protein Inhibitor-loaded Silica-coated Fe_3O_4 Nanoparticles (HSPI&Fe_3O_4@SiNPs)

Firstly, the Fe_3O_4 NPs were synthesized according to the literature procedure [1]. Briefly, an aqueous solution of $FeCl_3$ (2 mL, 1 mol/L) and $FeCl_2$ (0.5 mL, 2 mol/L, Sigma-Aldrich) in 2 mol/L HCl was mixed and added into diluted NH_3 solution (25 mL, 0.7 mol/L) and stirred for 30 min. Then, the precipitate was isolated by magnetic decantation and centrifugation. Solutions of Heat Shock Protein Inhibitor (HSPI, Selleckchem) were prepared by dissolving in PSB. Silica coating on the Fe_3O_4 NPs was accomplished by a water-in-oil reverse micelle method [2]. Typically, 350 μL Fe_3O_4 nanoparticles (2.0 mg/mL) and 50 μg/mL HSPI were dispersed into 7.7 mL cyclohexane, 2 mL Triton X-100, and 1.6 mL hexanol and stirred for 30 min to generate the microemulsion system. Then, 40 μL tetraethoxy orthosilicate (TEOS) was added to the mixture, followed by the addition of 100 μL aqueous ammonia for the TEOS hydrolysis under stirring for 24 h. After that, 50 μL tetraethoxy orthosilicate (TEOS) and 50 μL 3-aminopropyl triethoxysilane (APTS)/50 μL carboxyethylsilane triol sodium salt (CETS) were added to the mixture for another 24 h stirring. Finally, acetone was added to destabilize the microemulsion system. The HSPI-loaded Fe_3O_4@SiNPs were isolated *via* centrifugation and washed in sequence with ethanol and D.I. water for purification. All the reagents were obtained from Sigma-Aldrich.

Conjugation of Specific Antibody with HSPI-loaded Fe_3O_4@SiNPs

For specific antibody conjugation, the carboxyl functional groups were activated by 1-Ethyl-3-(3-dimethyllaminopropyl)carbodiimide hydrochloride (EDC) and N-Hydroxysuccinimide (NHS) in 2-(N-morpholino)ethanesulfonic acid (MES) buffer for 30 min. The MES buffer was then replaced by phosphate-buffered saline (PBS) (pH 7.4), and then 1 μM PE-labeled anti-CD20 (Invitrogen) was added into the HSPI-loaded Fe_3O_4@SiNPs suspension and stirred for 4 h in the dark. The resulting multifunctional nanoparticles with magnetic, fluorescence, and targeting properties were purified by washing with D.I. water and centrifugation, following by diluted in

100 μL PBS buffer and then were utilized for *in vitro* and *in vivo* experiments.

Characterization of Multifunctional Nanoparticles

Morphology of the MNPs was examined by field emission scanning electron microscope (FE-SEM, Philips XL30 Esem-FEG) and transmission electron microscope (TEM, Philips Tecnai 12 BioTWIN). The particle size and Zeta potentials were determined by a Malvern Zetasizer NanoZS instrument (Malvern, NanoZS). Magnetic properties were measured with a vibrating sample magnetometer (VSM, Model 1600, Digital Measurement System, Newton, MA).

The heat generation capabilities of the MNPs were measured using a 5 cm diameter 8-turn induction coil powered by a 3 kW alternating magnetic field (AMF) generator (SPG-06A, Shenzhen Shuangping Power Supply Technologies Co. Ltd.). Four differentiation samples (PBS, SiNPs, Fe_3O_4@SiNPs, and CD20-Fe_3O_4@SiNPs) were exposed to the AMF for 30 min. The frequency was kept constant at 350 kHz and the 21 kA/m field strength, and the temperature was monitored by using a thermometer immersed in a test tube containing 1 mg/ml of NPs solution.

***In vitro* Anti-cancer Drug Loading and Release**

For HSPI loading, 50 μg/mL HSPI were dispersed into the reaction solution. UV-vis spectrophotometer (PerkinElmer, PE Lamda 750, USA) in the range of 200-800 nm was used to calculate the loading amount of HSPI at 350 nm by the subtraction of free HSPI in the washing solution from the original amount. The encapsulation efficiency (EE) was calculated by using the following formula:

$$EE\ (\%) = [m_{(total\ HSPI)} - m_{(HSPI\ in\ supernatant)}] / m_{(total\ HSPI)} \times 100$$

The loading efficiency (LE) was evaluated by using the following formula:

$$LE\ (\%) = [m_{(total\ HSPI)} - m_{(HSPI\ in\ supernatant)}] / [m_{(loaded\ HSPI)} + m_{(carrier)}] \times 100$$

For drug release, 1 mL medium dispersed-HSPI-loaded Fe_3O_4@SiNPs was placed in a dialysis bag (weight cut-off of 10 kDa), and then immersed in 9 mL PBS and kept in an AMF at a constant temperature. Samples (300 mL) were periodically collected and the same volume of fresh medium was added. The amount of released HSPI was analyzed *via* UV-Visible spectrophotometry (U-3900, Hitachi) and the

concentration-absorbance standard equation.

Lung Cancer Stem Cell Culture

Human lung cancer stem cell line (LCSC) originated from human small cell lung cancer tissue was purchased from Celprogen (Cat# 36107-34, Celprogen, USA). Cell culture completed medium, 0.05% Trypsin-EDTA, and culture plates were purchased from Celprogen. Antibiotic solution (penicillin and streptomycin) were purchased from Invitrogen. LCSCs were maintained in a serum-free medium (DMEM/F12) supplemented with 2% B27, 10 ng/ml bFGF and EGF, 1% N2, and 1% antibiotic solution and cultured in a humidified atmosphere containing 5% CO_2 at 37 ^{o}C. The differentiated LCSCs (dLCSCs) were obtained by culturing LCSCs in the completed medium with 10% fetal bovine serum (FBS). The fresh medium was replaced ever three days of culture.

Immunofluorescence Staining for Stemness Analysis of LCSCs

The stemness of LCSCs was examined by immunofluorescence staining of surface makers and stemness markers. Briefly, LCSCs were cultured in 24-well plates were fixed with a solution of 4% paraformaldehyde in PBS for 30 min at 4°C, washed in PBS, treated with PBS/5% BSA for 1 h at room temperature and then stained with primary antibodies (Invitrogen) at room temperature for 1 h. The primary antibodies used were anti-human CD20 PE conjugated, anti-human CD15 FITC conjugated, anti-human ABCG APC conjugated. For analysis of stemness marker Oct4 expression, cells were fixed in 4% paraformaldehyde/PBS for 10 min, permeabilized with 0.1% Triton X-100 in PBS for 5 min, blocked with 3% BSA/PBS for 30 min, and then incubated with anti-human Oct4 FITC conjugated (Invitrogen) at room temperature for 1 h. The nuclei were stained with DAPI. Cells were then washed as described above and observed under the Laser-scanning confocal microscope (Leica SPE).

Tumor Sphere Formation Assay

LCSCs (3rd and 10th generation) were plated at a density of 10,000 cells/well in 6-well nonadherent plates (Corning Inc.) in DMEM/F12 cell medium, supplemented with human EGF (10 ng/mL, Invitrogen), N2 (1% v/v, Invitrogen), and human bFGF (10

ng/mL, Invitrogen). Fresh aliquots of EGF and bFGF were added every other day, and the spheres were collected at day 12 following gravity-based sedimentation and/or low-speed centrifugation to remove any remaining single cells. Spheres were then washed once with PBS, followed by gentle resuspension in cell detachment solution and constant trituration for at least 20 min to obtain single cells. The dissociated single cells of the primary spheres were washed at least three times with the medium and then plated for sequential sphere-formation for an additional 12 days.

In vitro Cytotoxicity of Multifunctional Nanoparticles

To study the cytotoxicity of designed nanoparticles, LCSCs were seeded at 5×10^3 cells/well in a 96-well plate, pre-incubated for 24 h, then incubated with Fe_3O_4@SiNPs (free HSPI), HSPI or HSPI-loaded Fe_3O_4@SiNPs (HSPI&Fe_3O_4@SiNPs) for 24 h at concentrations ranging from 10 to 500 μg/mL. The medium was moved and the cells were washed with PBS three times. A volume of 10 μL 3-(4, 5-Dimethylthiazol-2-yl)-2, 5-diphenyltetrazolium bromide (MTT, Invitrogen) at a concentration of 0.5 mg/mL was added in each well. Following incubation for 4 h, formazan crystals formed were dissolved in 150 mL dimethylsulfoxide (DMSO, Sigma-Aldrich) and absorbance was measured at 570 nm with a reference wavelength of 630 nm using a microplate reader (MD VersaMax). Untreated cells in medium were used as control. All experiments were carried out with five replicates.

Hemolysis Assay and Biochemical Analysis

The whole blood was centrifuged at 3000 rpm for 5 min to harvest the red blood cells (RBCs), and then washed three times with saline and diluted with PBS to 1 mL. The hemolytic effect of NPs was evaluated by incubating with 50 μL CD20-HSPI&Fe_3O_4@SiNPs (final concentration 1 mg/mL) at 37°C for 1 h. The final volume of the hemolysis assay in all experiments was 1.0 mL. The same amount of RBC suspension incubated with 500 μL PBS and water was used as the negative and positive control, respectively. The supernatant absorbance was tested at 540 nm by microplate reader. The positive and negative group value should be 0.8 ± 0.3 and less than 0.03, respectively. The followed equation: Hemolytic rate (%) = [(OD_{sample} – OD

$_{negative}$)/ (OD $_{positive}$ − OD $_{negative}$)] × 100% was used to calculated the percentage of hemolysis. The blood of LCSC-tumor bearing mice (after 40 days treatment) was collected, and then the serum biochemical markers tested. Liver function was determined by the levels of AST, ALT, and ALP. The renal function was measured by the levels of Cr and UA.

Tumor Xenograft and Organ Tissues Staining

To further investigate the therapeutic effects of MNPs on tumor-bearing mice treated by retro-orbital sinus injection, the tumors were excised for immunohistochemisical (IHC) analysis on day 40 post-injection. Meanwhile, organs were collected for studying the side effects of MNPs on mice by IHC analysis. After fixation, tissues were sectioned at 5 μm thickness after fixing in formalin and embedding in paraffin, and then stained with hematoxylin and eosin (H&E). The IHC images were taken under a microscope (CX41, Olympus). The significant treatment efficiency of MNPs was evaluated by immunofluorescence staining. Tumor xenograft sections were incubated with anti-CD20/PE to at 37 °C for 1 h, and then examined by a confocal microscope.

Results

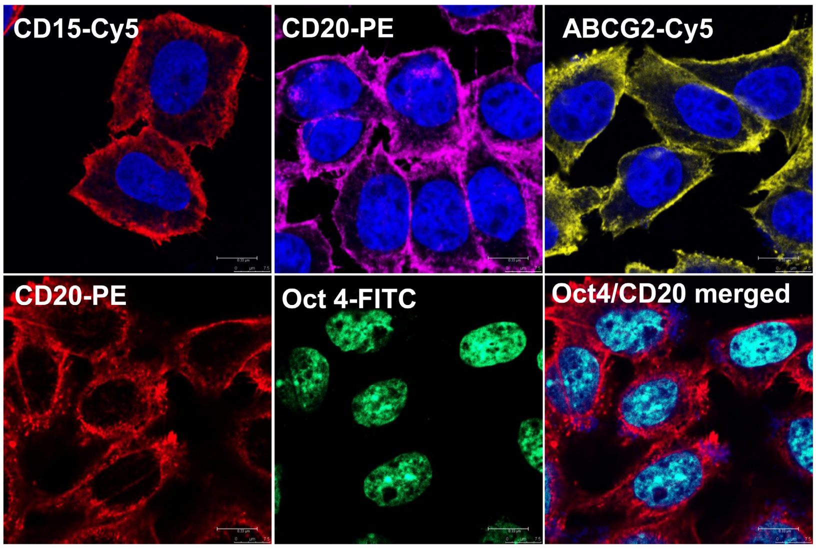

Figure S1. A lung cancer stem cell model with specific expression of CD20, CD15, ABCG2, and a stemness marker Oct4 (blue color: nucleus).

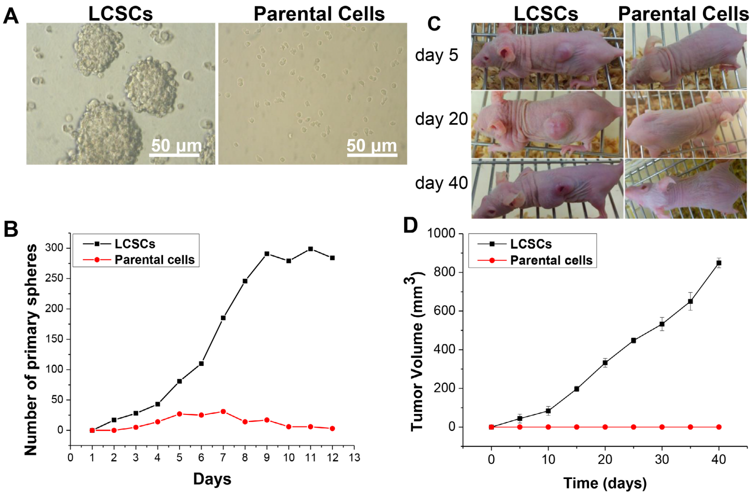

Figure S2. *In vitro* and *in vivo* tumourigenicity of LCSCs. (A) Tumor sphere formation; (B) Quantitative data of tumor sphere; (C) Tumorigenic capacity of LCSCs; (D) Quantitative data of tumor formation *in vivo*.

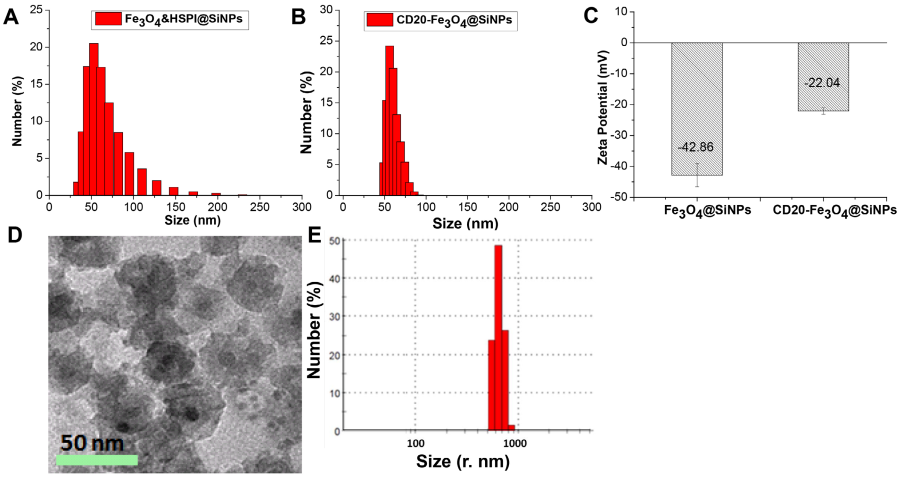

Figure S3. Size distribution of (A) Fe_3O_4&HSPI@SiNPs and (B) CD20-Fe_3O_4&HSPI@SiNPs. (C) Zeta potential of NPs determined by dynamic light scattering (DLS). TEM image (D) and size distribution (E) of the MNPs after AMF treatment.

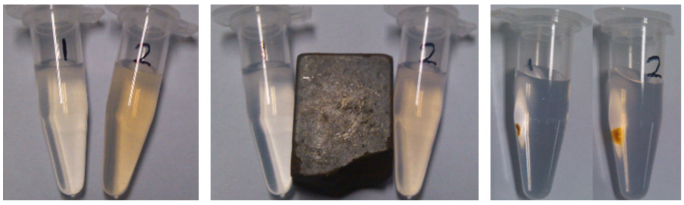

Figure S4. Magnetic property of the MNPs. Magnetic nanoparticles suspended in a liquid are attracted to a magnet.

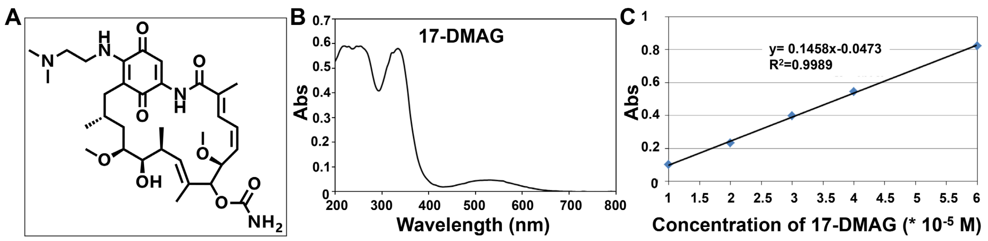

Figure S5. The structure and encapsulation efficiency of 17-DMAG. (A) The structure of 17-DMAG (HSPI); (B) UV-absorbance of 17-DMAG; (C) Standard curve of drug loading.

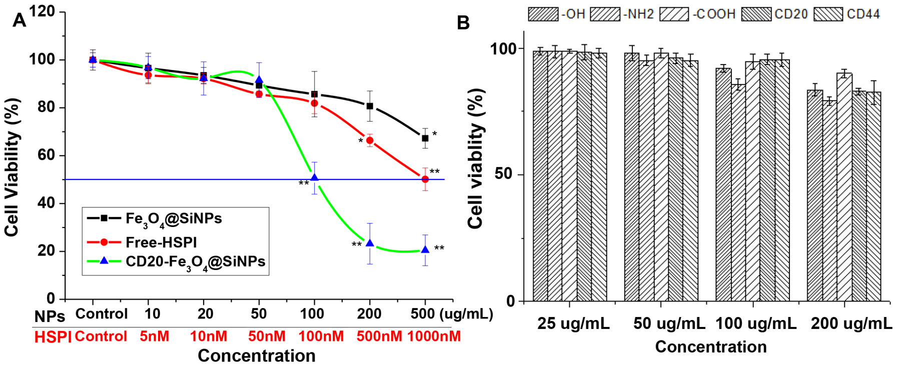

Figure S6. *In vitro* cell cytotoxicity of SiNPs with different modification (A) and different functional groups (B).

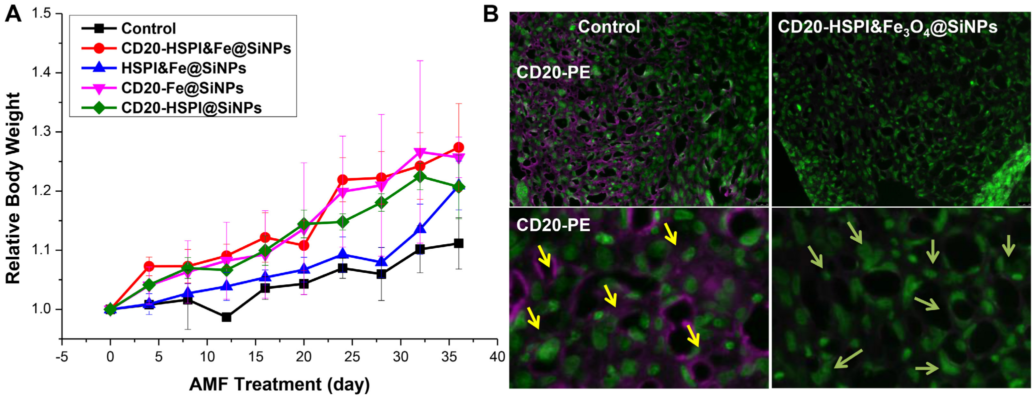

Figure S7. (A) Relative body weight of the mice after treatment with various NPs. (B) Immunohistochemically stained with PE-conjugated CD20 antibodies.

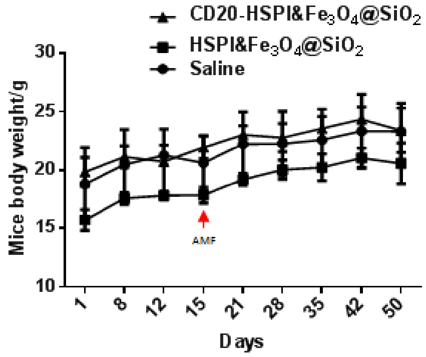

Figure S8. Plot of body weight of the mice after treatment with various NPs.

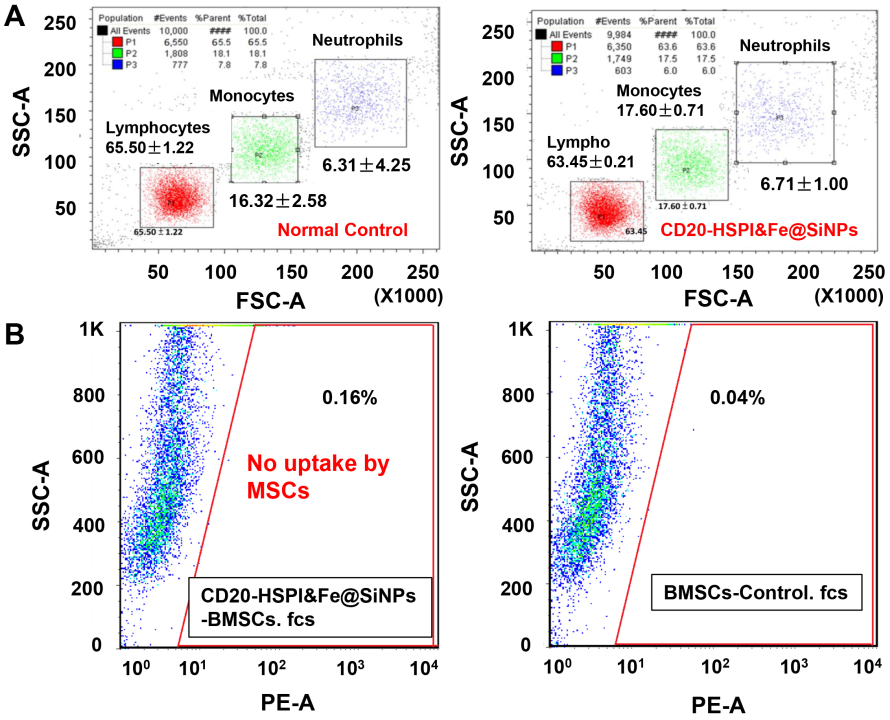

Figure S9. (A) Flow cytometry analysis of lymphocytes, monocytes and macrophages, and neutrophils in white blood cell populations by forward and side scatter analysis. (B) Flow cytometry analysis of uptake of MNPs in mesenchymal stem cells.

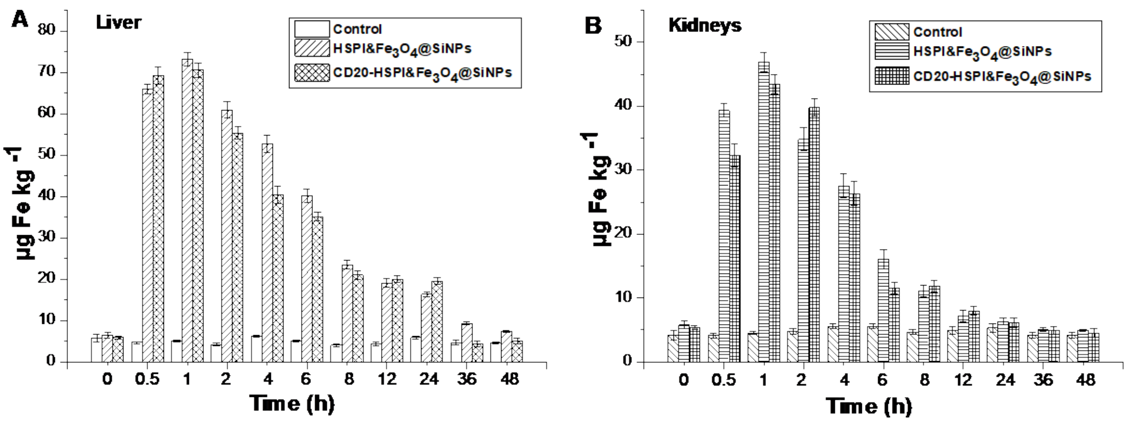

Figure S10. Fe element content in liver (A) and kidneys (B) showed the long-term accumulation of the MNPs in mouse after 48 h *i.v.* injection.

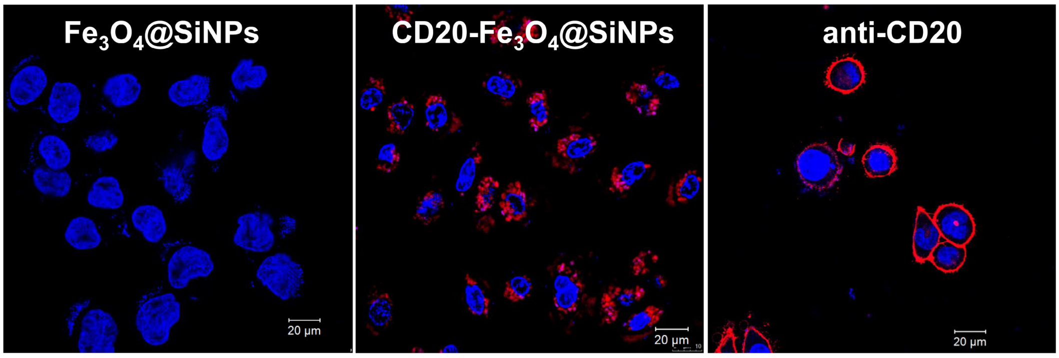

Figure S11. Cellular uptake of Fe_3O_4@SiNPs, CD20-Fe_3O_4@SiNPs, and anti-CD20 by LCSCs.

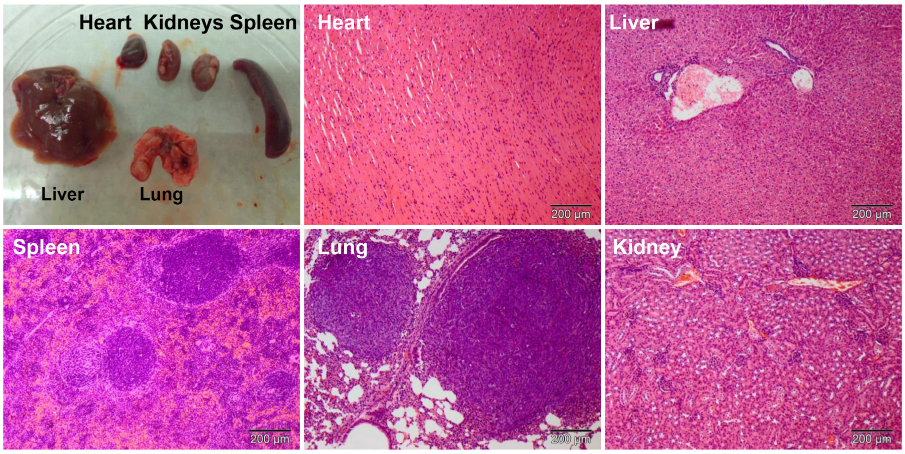

Figure S12. Establishment of LCSC lung metastastic mouse model. Macroscopic view of mouse organs and H&E staining for organs tissue section.

Table S1. Changes of Biochemical in the Serum of Mice (n=8).

Groups	Cr(μmol/L)	UA(μmol/L)	AST(U/L)	ALT(U/L)	ALP(U/L)
**Control**	13.75±2.47	433.08±9.23	199.50±6.74	40.23±4.56	47.00±3.74
**MNPs**	14.32±2.46	469.54±8.78	207.78±9.15	43.19±6.77	49.00±5.01

References

1. Kalantari K, Ahmad MB, Shameli K, Hussein MZB, Khandanlou R, Khanehzaei H. Size-controlled synthesis of Fe_3O_4 magnetic nanoparticles in the layers of montmorillonite. J Nanomater. 2014; 2014: 1-9.
2. Ding HL, Zhang YX, Wang S, Xu JM, Xu SC, Li GH. Fe_3O_4@SiO_2 core/shell nanoparticles: the silica coating regulations with a single core for different core sizes and shell thicknesses. Chem Mater. 2012; 24: 4572-80.
